# The N-Myc-responsive lncRNA MILIP promotes DNA double-strand break repair through non-homologous end joining

**DOI:** 10.1073/pnas.2208904119

**Published:** 2022-11-29

**Authors:** Pei Lin Wang, Liu Teng, Yu Chen Feng, Yi Meng Yue, Man Man Han, Qianqian Yan, Kaihong Ye, Cai Xia Tang, Sheng Nan Zhang, Teng Fei Qi, Xiao Hong Zhao, Ting La, Yuan Yuan Zhang, Jin Ming Li, Bin Hu, Dengfei Xu, Shundong Cang, Li Wang, Lei Jin, Rick F. Thorne, Yuwei Zhang, Tao Liu, Xu Dong Zhang

**Affiliations:** ^a^Translational Research Institute, Henan Provincial and Zhengzhou City Key laboratory of Non-coding RNA and Cancer Metabolism, Henan International Join Laboratory of Non-coding RNA and Metabolism in Cancer, Henan Provincial People’s Hospital, Academy of Medical Sciences, Zhengzhou University, Zhengzhou, Henan 450053, China; ^b^School of Medicine and Public Health, The University of Newcastle, Newcastle, NSW 2308, Australia; ^c^School of Biomedical Sciences and Pharmacy, The University of Newcastle, Newcastle, NSW 2308, Australia; ^d^Department of Oncology and Oncology Radiotherapy, Henan Provincial People's Hospital, Henan 450003, Zhengzhou, China; ^e^School of Basic Medical Sciences, Zhengzhou University, Zhengzhou, Henan 450003, China; ^f^Henan Key Laboratory of Stem Cell Differentiation and Modification, Henan Provincial People's Hospital, Henan University, Zhengzhou, Henan 450003, China; ^g^Children's Cancer Institute Australia for Medical Research, University of New South Wales, Sydney, NSW 2750, Australia

**Keywords:** lncRNA, N-Myc, *MYCN*, DNA repair, neuroblastoma

## Abstract

The protooncoprotein N-Myc, which is overexpressed in approximately 25% of neuroblastomas as the consequence of *MYCN* gene amplification, has long been postulated to regulate DNA double-strand break (DSB) repair in neuroblastoma cells, but experimental evidence of this function is presently scant. Here, we show that N-Myc transcriptionally activates the long noncoding RNA MILIP to promote nonhomologous end-joining (NHEJ) DNA repair through facilitating Ku70–Ku80 heterodimerization in neuroblastoma cells. High MILIP expression was associated with poor outcome and appeared as an independent prognostic factor in neuroblastoma patients. Knockdown of MILIP reduced neuroblastoma cell viability through the induction of apoptosis and inhibition of proliferation, retarded neuroblastoma xenograft growth, and sensitized neuroblastoma cells to DNA-damaging therapeutics. The effect of MILIP knockdown was associated with the accumulation of DNA DSBs in neuroblastoma cells largely due to decreased activity of the NHEJ DNA repair pathway. Mechanistical investigations revealed that binding of MILIP to Ku70 and Ku80 increased their heterodimerization, and this was required for MILIP-mediated promotion of NHEJ DNA repair. Disrupting the interaction between MILIP and Ku70 or Ku80 increased DNA DSBs and reduced cell viability with therapeutic potential revealed where targeting MILIP using Gapmers cooperated with the DNA-damaging drug cisplatin to inhibit neuroblastoma growth in vivo. Collectively, our findings identify MILIP as an N-Myc downstream effector critical for activation of the NHEJ DNA repair pathway in neuroblastoma cells, with practical implications of MILIP targeting, alone and in combination with DNA-damaging therapeutics, for neuroblastoma treatment.

## Significance

Here, we report that the lncRNA MILIP is transcriptionally regulated by N-Myc and functions to promote DNA double-strand break repair in neuroblastoma cells. MILIP is distinguished from other lncRNAs implicated in DNA repair through its scaffolding role in Ku complex formation, a requisite step to initiate the nonhomologous end-joining pathway. Our findings substantiate the long-postulated role of N-Myc in regulating DNA repair in neuroblastoma cells and reveal the functional importance of MILIP in cell survival, proliferation, and resistance to genotoxic stress, with practical implications of MILIP targeting, alone and in combination with DNA-damaging therapeutics, for neuroblastoma treatment.

Neuroblastoma is the most frequent solid tumor in young children, representing about 15% of all childhood cancer-related deaths (1). Amplification of the protooncogene *MYCN* and subsequent overexpression of the oncoprotein N-Myc occurs in approximately 25% of neuroblastomas and correlates with poor outcomes (1, 2). N-Myc plays a profound role in regulating diverse cellular processes (1, 2), but nonetheless, the role of N-Myc in regulating DNA double-strand break (DSB) repair has only begun to emerge. Noticeably, N-Myc was recently shown to prevent DNA DSB accumulation through resolving DNA transcription–replication conflicts (TRCs) (3). Moreover, several genes involved in DNA repair are known to be deregulated in *MYCN*-amplified neuroblastomas (4).

In response to DNA damage, cells activate the DNA damage response to identify and repair damaged DNA (5, 6). However, upon excessive damage to DNA and/or when repair is ineffective, cells commit suicide or alternatively repair errors resulting in genetic mutations (5, 6). Although DSBs can be repaired through homologous recombination (HR), an error-free mechanism that only operates at the S and G2 phases (6), the preferred repair pathway in cancer cells is nonhomologous end joining (NHEJ), an error-prone mechanism acting throughout the cell cycle (6, 7). NHEJ is initiated with the recognition of DSB ends by the Ku complex, the heterodimer of Ku70 and Ku80 (6–9). This leads to the recruitment of other proteins necessary for processing and ligation of the broken ends, including DNA-dependent protein kinase catalytic subunit (DNA-PKcs), Artemis, and DNA ligase IV (10, 11). DSBs can also be repaired by the alternative NHEJ pathway, a less-efficient mechanism independent of NHEJ (10, 12).

There is increasing appreciation of the role of long noncoding RNAs (lncRNAs) in cancer development, progression, and treatment resistance (13). In particular, a growing number of lncRNAs have been linked to DNA damage repair (14–16). For example, the lncRNA LINP1 facilitates the interaction of Ku80 with DNA-PKcs in breast cancer and cervical cancer cells (14), the lncRNA NIHCOLE promotes the stable multimeric complexes of NHEJ factors in hepatocellular carcinoma cells (15), and the lncRNA LRIK interacts with the Ku complex to promote its binding to DSB sites (16). Moreover, several p53-regulated lncRNAs such as DINO and MALAT1 are involved in the regulation of DSB repair (17, 18).

Here, we show that MILIP, an N-Myc-responsive lncRNA, promotes the NHEJ pathway through facilitating Ku70–Ku80 heterodimerization and is involved in the resistance to DNA-damaging therapeutics in neuroblastoma cells. Moreover, revealing the therapeutical potential of targeting MILIP, we establish its cooperation with genotoxic stress to inhibit neuroblastoma growth in vivo. Thus, MILIP targeting represents a potential avenue for the development of improved neuroblastoma treatment.

## Results

### N-Myc Regulates MILIP Expression in Neuroblastoma and High MILIP Expression in Neuroblastoma Tissues is Associated with Poor Patient Outcome.

**Fig. 1.**
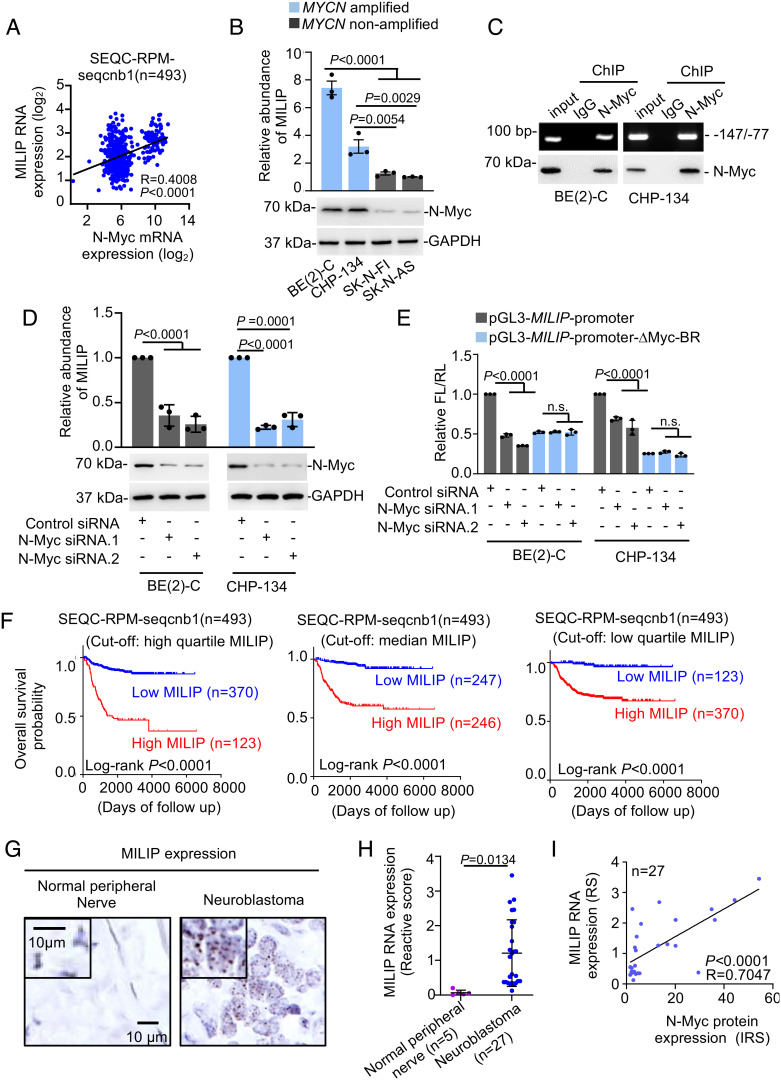
N-Myc regulates MILIP expression that is associated with poor patient outcome. (*A*) Regression analysis of the relationship between MILIP and N-Myc mRNA expression (RPM) in the RNA-seq SEQC-RPM-seqcnb1 neuroblastoma dataset. (*B*) MILIP expression in neuroblastoma cell lines with or without *MYCN* amplification. (*C*) N-Myc (*Lower* panel) bound to the *MILIP* promoter (*Upper* panel). (*D*) N-Myc knockdown down-regulated MILIP expression. (*E*) Luciferase reporter assays showing that the transcriptional activity of a *MILIP* promoter reporter construct containing the Myc-BR was reduced by N-Myc knockdown, whereas a *MILIP* promoter reporter construct with the Myc-BR deleted displayed decreased activity that was not affected by N-Myc knockdown. (*F*) Kaplan–Meier analysis of the probability of patient overall survival (OS). (*G*) Representative microscopic photographs of ISH analysis of MILIP expression in FFPE tissues (n = 27 tumors). (*H*) Quantitation of MILIP expression as detected in *G*. (*I*) Regression analysis of the relationship between MILIP expression (*G*) and N-Myc protein expression (*SI Appendix*, Fig. S2*C*). RS: reactive score; IRS: immunoreactive score. Data shown are mean ± SEM (*B*, bar charts in *D* and *E*) or representative (*C* and western blotting panels in *D*) of 3 independent experiments. One-way ANOVA followed by Tukey’s multiple comparison test (*B*, *D*, and *E*) or two-tailed Student’s *t* test (*H*).

Through interrogating the RNA-sequencing (RNA-seq) SEQC-RPM-seqcnb1 neuroblastoma dataset acquired from the R2 Genomics Analysis and Visualization Platform (R2, http://r2.amc.nl), we identified a panel of lncRNAs with expression levels positively correlated with N-Myc mRNA expression (*SI Appendix*, Fig. S1*A*). Among them was MILIP that is activated by c-Myc in other types of cancers (Fig. 1*A* and *SI Appendix,* Fig. S1*A*) (19). This correlative relationship between MILIP and N-Myc mRNA was similarly observed in additional neuroblastoma datasets (*SI Appendix,* Fig. S1*B*). In accordance, MILIP was expressed at higher levels in *MYCN*-amplified than *MYCN*-nonamplified neuroblastomas (*SI Appendix,* Fig. S1*C*). Cursory screening of neuroblastoma cell lines showed that MILIP levels were also positively associated with N-Myc protein levels (Fig. 1*B*). Absolute quantitation revealed that there were 286 MILIP molecules per BE(2)-C cell (*MYCN*-amplified) compared with 128 MILIP molecules per SK-N-FI cell (*MYCN*-nonamplified) (*SI Appendix,* Fig. S1*D*). These observations point to a regulatory relationship between N-Myc and MILIP expression.

The *MILIP* gene promoter contains an E-box motif that is the consensus binding region of Myc proteins (Myc-BR) (*SI Appendix,* Fig. S1*E*) (19, 20). Indeed, N-Myc coprecipitated the *MILIP* promoter as shown in ChIP assays (Fig. 1*C*). SiRNA knockdown of N-Myc reduced MILIP expression along with the lncRNA PVT1 known to be regulated by N-Myc in *MYCN*-amplified BE(2)-C and CHP-134 cells (Fig. 1*D* and *SI Appendix,* Fig. S1*F*) (21), whereas transfection with an N-Myc overexpression construct up-regulated MILIP in *MYCN*-nonamplified SK-N-FI and SK-N-AS cells (*SI Appendix,* Fig. S1*G*). Moreover, N-Myc knockdown diminished, whereas N-Myc overexpression enhanced transcriptional activity of luciferase reporters of the *MILIP* promoter containing the intact Myc-BR (Fig. 1*E* and *SI Appendix,* Fig. S1*H*). Conversely, neither N-Myc knockdown nor overexpression further altered the reduced reporter activity of the *MILIP* promoter with the Myc-BR deleted (∆Myc-BR) (Fig. 1*E* and *SI Appendix,* Fig. S1*H*). Therefore, N-Myc transcriptionally activates MILIP through the Myc-BR in neuroblastoma cells. Noticeably, neither knockdown nor overexpression of MILIP altered N-Myc expression levels (*SI Appendix,* Fig. S1 *I* and *J*), indicating that MILIP does not have a role in regulating N-Myc expression.

Kaplan–Meier survival curves showed that high MILIP expression in human neuroblastoma tissues was associated with poor progression-free survival (PFS) and OS in the 493 patients included in the R2 SEQC-RPM-seqcnb1 neuroblastoma dataset using the high quartile, median, or low quartile of MILIP levels as the cutoff points (Fig. 1*F* and *SI Appendix,* Fig. S2*A*). Similarly, when the high-quartile MILIP expression was used as the cutoff point, high MILIP levels were associated with poor PFS and OS of patients in the human neuroblastoma tissue gene expression Versteeg dataset (*SI Appendix*, Fig. S2*B*). Multivariable Cox regression analysis of the SEQC-RPM-seqcnb1 neuroblastoma dataset revealed that high MILIP expression was associated with PFS and OS independently of age at diagnosis, disease stage, and the amplification status of the *MYCN* gene, well-established prognostic markers of neuroblastoma patients (Table 1) (1, 22). Therefore, high MILIP expression in neuroblastoma tissues is potentially an independent prognostic factor of patient outcome.

**Table 1.** Multivariable Cox regression analysis of factors prognostic of outcome in 493 neuroblastoma patients^*^, ^†^

Factors	Progression-free survival		Overall survival	
	HR (95%CI)	*P* value	HR (95%CI)	*P* value
High MILIP expression (median as the cutoff)	1.63 (1.13–2.37)	0.010	2.66 (1.44–4.91)	0.022
Age > 18 mo	1.59 (1.11–2.26)	0.011	2.50 (1.45–4.31)	0.001
Stages 3 and 4^‡^	2.80 (1.87–4.20)	6.2E–7	5.00 (2.35–10.60)	2.8E–5
MYCN amplification	1.51 (1.08–2.13)	0.017	2.72 (1.81–4.10)	2.0E–6

^*^Data were extracted from the RNA-sequencing SEQC-RPM-seqcnb1 dataset.

^†^MILIP expression levels were considered high or low in relation to the median level of expression in all tumors analyzed. Hazard ratios were calculated as the antilogs of the regression coefficients in the proportional hazards regression. Multivariable Cox regression analysis was performed following the inclusion of the four above-listed factors into the Cox regression model, and the *P* value was obtained from the two-sided log-rank test.

^‡^Tumor stage was categorized as favorable (International Neuroblastoma Staging System stages 1, 2, and 4S) or unfavorable (International Neuroblastoma Staging System stages 3 and 4).

Using in situ hybridization (ISH) analysis, we confirmed that MILIP was readily detected in neuroblastoma cells with primarily nuclear localization in human neuroblastoma tissues (Fig. 1 *G* and *H*). Moreover, MILIP levels were correlated with the levels of N-Myc protein detected by immunohistochemistry (IHC) (Fig. 1*I* and *SI Appendix,* Fig. S2*C*). Nuclear localization of MILIP was also observed in cultured neuroblastoma cells (*SI Appendix,* Fig. S2 *D* and *E*). There were no significant differences in the expression of MILIP between neuroblastomas stratified according to their gender, median age at diagnosis, and stages (*SI Appendix,* Table S1), suggesting that MILIP upregulation is commonly an early event during the development of neuroblastoma.

### MILIP Promotes Neuroblastoma Tumorigenicity.

**Fig. 2.**
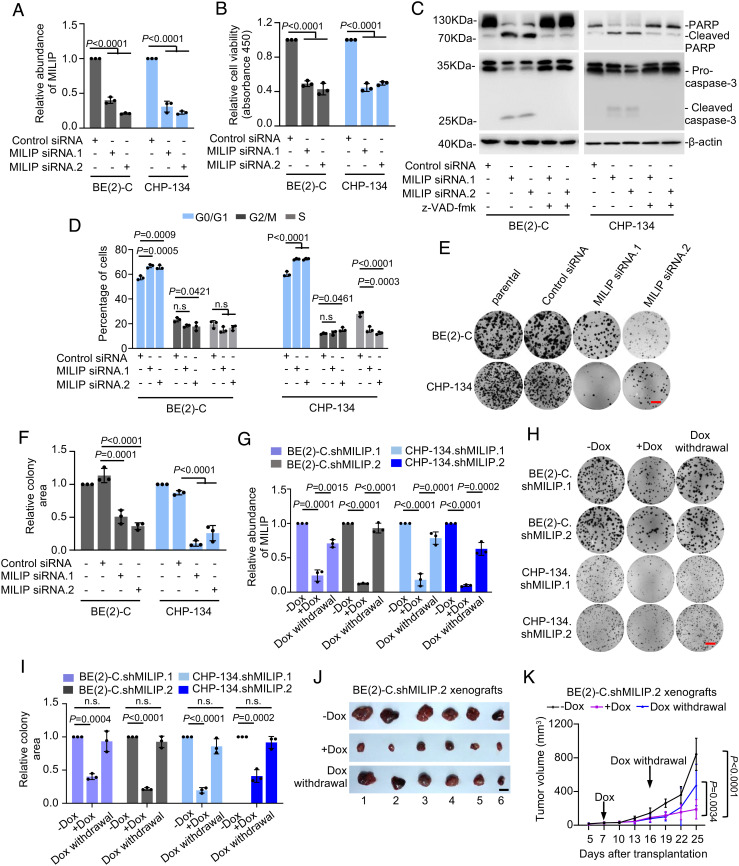
MILIP promotes neuroblastoma tumorigenicity. (*A*–*E*) MILIP knockdown (*A*) reduced cell viability (*B*), induced caspase-3 activation and PARP cleavage (*C*), caused G0/G1 cell cycle arrest (*D*), and reduced clonogenicity (*E*). (*F*) Quantitation of relative clonogenicity as shown in *E*. (*G* and *H*) MILIP expression (*G*) and the clonogenicity in cells with induced MILIP knockdown were recovered upon withdrawal of doxycycline (Dox). (Scale bar, 1 cm.) (*I*) Quantitation of relative clonogenicity as shown in *H*. (*J* and *K*) Photographs (*J*) and growth curves (*K*) of BE(2)-C.shMILIP.2 xenografts in nu/nu mice with or without cessation of Dox treatment. (Scale bar, 1 cm.) Data shown are mean ± SEM (*A*, *B*, *D*, *F*, *G*, and *I*) or representative (*C*, *E*, and *H*) of 3 independent experiments. One-way ANOVA followed by Tukey’s multiple comparison test.

SiRNA knockdown of MILIP inhibited BE(2)-C and CHP-134 cell viability (Fig. 2). This was associated with induction of apoptosis as shown by caspase-3 activation and cleavage of the caspase-3 substrate PARP (Fig. 2*C*) (23). However, the addition of the general caspase inhibitor z-VAD-fmk only partially inhibited the reduced cell viability following MILIP knockdown (*SI Appendix,* Fig. S3*A*), although it abolished caspasae-3 activation and PARP cleavage (Fig. 2*C*), indicative of the involvement of other mechanisms. Indeed, MILIP knockdown also triggered, although moderately, G0/G1 cell cycle arrest (Fig. 2*D*). The effect of MILIP knockdown on neuroblastoma cell viability was also evident in clonogenic assays (Fig. 2 *E* and *F*). Conversely, MILIP overexpression enhanced, although moderately, proliferation of SK-N-FI and SK-N-AS cells (*SI Appendix,* Fig. S3 *B*–*D*),

To facilitate further investigations, we established BE(2)-C and CHP-134 sublines, BE(2)-C.shMILIP and CHP-134.shMILIP, respectively, with MILIP conditionally knocked down in response to doxycycline (Dox). The addition of Dox reduced MILIP expression and inhibited cell viability and clonogenicity (Fig. 2 *G*–*I* and *SI Appendix,* Fig. S3*E*). After Dox was withdrawn, MILIP levels recovered, and clonogenicity was restored (Fig. 2 *G*–*I*). Treatment of nu/nu mice bearing neuroblastoma xenografts established by subcutaneous transplantation of BE(2)-C.shMILIP cells with Dox retarded tumor growth (Fig. 2 *J* and *K* and *SI Appendix,* Fig. S3 *F* and *G*), which was similarly associated with induction of apoptosis and reduced cell proliferation (*SI Appendix,* Fig. S3 *H–K*). Cessation of Dox treatment resulted in recovery of MILIP expression and regrowth of the tumors (Fig. 2 *J* and *K* and *SI Appendix,* Fig. S3 *F* and *G*). Thus, MILIP plays a role in promoting neuroblastoma cell survival, proliferation, and tumorigenicity.

### MILIP Facilitates Ku70–Ku80 Heterodimerization.

MILIP supports cell survival and proliferation through directly binding to and repressing p53 in some cancer cell types such as A549 lung adenocarcinoma cells (19). However, unlike A549 cells, p53 was not recovered with MILIP in RNA pull-down experiments conducted in wild-type p53-expressing CHP-134 cells (*SI Appendix,* Fig. S4*A*), indicating that the function of MILIP in neuroblastoma is independent of direct interactions with p53. Nevertheless, MILIP knockdown caused moderate upregulation of p53 (*SI Appendix,* Fig. S4 *B* and *C*), which was associated with phosphorylation of ataxia telangiectasia mutated (ATM) (pS1981), phosphorylation of checkpoint kinase 2 (CHK2) (pT68), and phosphorylation of p53 (pS15) in CHP-134 cells (*SI Appendix,* Fig. S4 *B* and *C*), signifying activation of the ATM/CHK2/p53 pathway in response to DSBs (24). Moreover, MILIP knockdown-induced apoptosis was inhibited, at least partially, in CHP-134 cells with p53 knockout using CRISPR/Cas9 genome editing (*SI Appendix,* Fig. S4 *D–G*). Together, these results suggest that MILIP protects neuroblastoma cells from DSBs and that p53 plays a role in induction of apoptosis in wild-type p53 neuroblastoma cells when MILIP is inhibited.

**Fig. 3.**
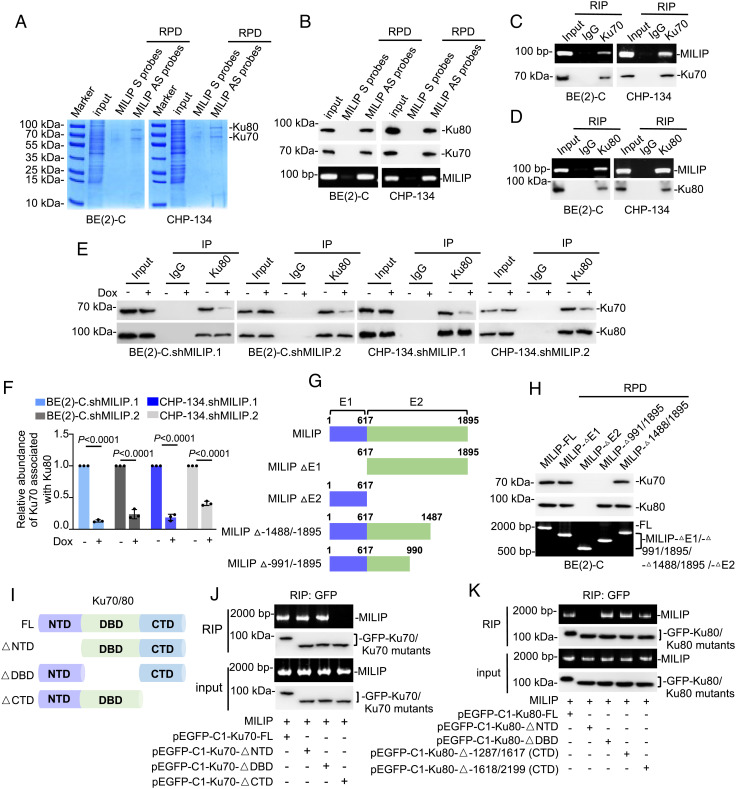
MILIP facilitates Ku70–Ku80 heterodimerization. (*A*) RPD followed by mass spectrometry analysis showing that Ku70 and Ku80 bound to MILIP (n = 2 independent experiments). S: sense; AS: antisense. (*B*) Endogenous Ku70 and Ku80 were copulled down by MILIP. (*C* and *D*) MILIP was coprecipitated with endogenous Ku70 (*C*) and Ku80 (*D*). (*E*) MILIP knockdown reduced the amount of endogenous Ku70 coprecipitated with Ku80. (*F*) Quantitation of the relative amount of Ku70 associated with Ku80 as shown in *E*. (*G*) Schematic illustration of full-length MILIP (MILIP-FL) and MILIP mutants used for deletion mapping. (*H*) MILIP Δ-1488/-1895 but not Δ-991/-1895 pulled down Ku70, whereas MILIP Δ-991/-1895 but not ΔE2 pulled down Ku80 in a cell-free system. (*I*) Schematic illustration of full-length Ku70 and Ku80 (Ku70-FL and Ku80-FL, respectively) and Ku70 or Ku80 mutants with its N terminus, the central DNA-binding domain, or C terminus deleted (K70/80-△NTD, K70/80-△DBD, or K70/80-△CTD). (*J*) RIP assays showing that in vitro–synthesized MILIP was coprecipitated with purified Ku70-△NTD and Ku70-△DBD but not Ku70-△CTD in a cell-free system. (*K*) RIP assays showing that in vitro–synthesized MILIP was coprecipitated with Ku80-△CTD and Ku80-△DBD but not Ku80-△NTD in a cell-free system. Data shown are mean ± SEM (*F*) or representative (*B*, *C*, *D*, *E*, *H*, *J*, and *K*) of 3 independent experiments. A two-tailed Student’s *t* test.

To further understand the mechanism responsible for MILIP-mediated promotion of neuroblastoma cell survival and proliferation, we analyzed the proteins that interact with MILIP using RNA pulldown (RPD) followed by mass spectrometry. This identified Ku70 as the most abundant protein that coprecipitated with MILIP, whereas Ku80 was also readily detected in the precipitates (Fig. 3*A* and *SI Appendix,* Table S2). The association of endogenous MILIP with Ku70 and Ku80 was confirmed using RNA pull-down and RIP assays (Fig. 3 *B*–*D*). Moreover, two-step RIP assays demonstrated that antibodies (Abs) against Ku80 precipitated Ku80 along with Ku70 and MILIP, and in the second-phase immunoprecipitation, anti-Ku70 Abs coprecipitated Ku80 and MILIP (*SI Appendix,* Fig. S4*H*), indicating that MILIP, Ku70, and Ku80 form an RNA–protein ternary complex. Strikingly, MILIP knockdown reduced, whereas MILIP overexpression increased, the interaction between Ku70 and Ku80 (Fig. 3 *E* and *F* and *SI Appendix,* Fig. S4 *I* and *J*). Therefore, MILIP interacts with Ku70 and Ku80 and promotes their heterodimerization. Treatment with DNase I did not affect the associations of MILIP with Ku70 and Ku80 (*SI Appendix*, Fig. S4*K*), indicating that the binding of these proteins to DSB ends is not necessary for their interaction with MILIP. Noticeably, neither knockdown nor overexpression of MILIP affected Ku70 and Ku80 expression (Fig. 3*E* and *SI Appendix,* Fig. S4*I*).

To define the region of MILIP responsible for its interaction with Ku70 and Ku80, we carried out deletion-mapping experiments with MILIP mutants transcribed in vitro (Fig. 3*G*). This analysis showed that MILIP fragment corresponding to exon 2 is required for the MILIP interaction with Ku70 and Ku80 (Fig. 3 *G* and *H*). Further incremental deletions revealed that Δ-991/-1895 but not Δ-1488/-1895 MILIP eliminated its binding to Ku70, whereas Δ-991/-1895 MILIP retained the ability to interact with Ku80 (Fig. 3 *G* and *H*). Thus, the fragments -991/-1487 and -617/-990 are responsible for the association of MILIP with Ku70 and Ku80, respectively. Deletion mapping with mutants of Ku70 showed that removing the C terminus of Ku70, but not other regions, abolished its association with MILIP (Fig. 3 *I* and *J*). Likewise, deletion of the N terminus of Ku80 inhibited its interaction with MILIP (Fig. 3 *I* and *K*). Therefore, the MILIP-binding fragments of Ku70 and Ku80 occur within their C and N terminus, respectively, and do not overlap with the central Ku70–Ku80 DNA-binding domains, which are known to mediate Ku70–K80 heterodimerization (25).

### MILIP Promotes NHEJ Activation.

**Fig. 4.**
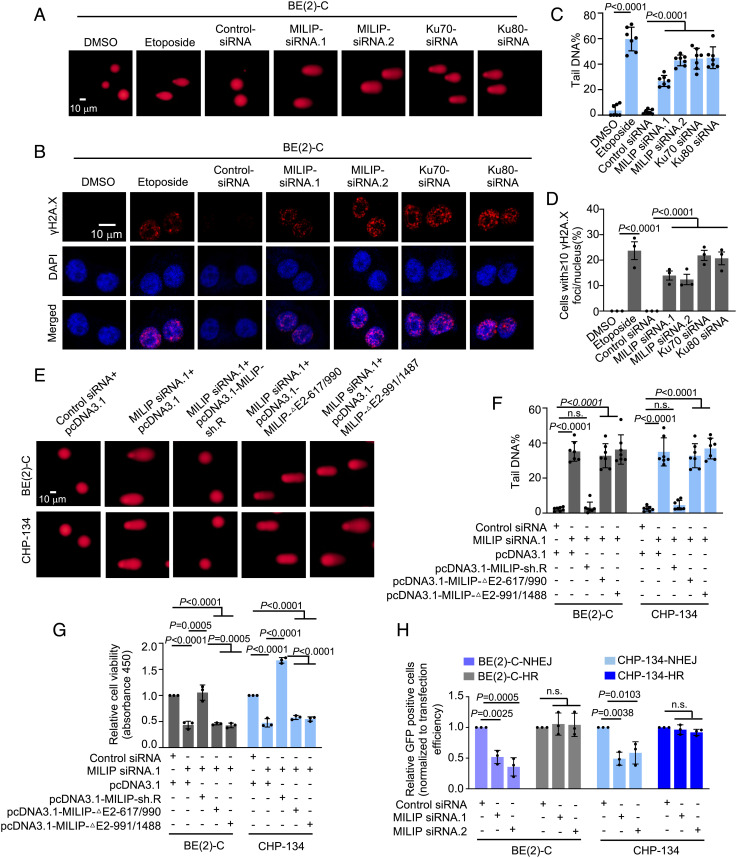
MILIP promotes the NHEJ pathway. (*A* and *B*) MILIP knockdown induced the appearance of comet tails (*A*) and the formation of γH2A.X foci (red) (*B*). (*C* and *D*) Quantitation of the relative tail DNA content of the comets and γH2A.X foci as shown in *A* (*C*) and *B* (*D*), respectively. (*E*) Introduction of MILIP-sh.R but not MILIP-△-617/-990 or MILIP-△-991/-1487 reduced the comet tails caused by MILIP knockdown. (*F*) Quantitation of the relative tail DNA content as shown in *E*. (*G*) Introduction of MILIP-sh.R but not MILIP-△-617/-990 or MILIP-△-991/-1487 diminished the inhibition of cell viability caused by knockdown of endogenous MILIP. (*H*) MILIP knockdown caused reductions in NHEJ but not HR activity measured using NHEJ and HR GFP reporters, respectively. Data shown are mean ± SEM (*C*, *D*, *F*, *G*, and *H*) or representative (*A*, *B*, and *E*) of 3 independent experiments. One-way ANOVA followed by Tukey’s multiple comparison test.

Having demonstrated that MILIP facilitates Ku70–Ku80 heterodimerization that is essential for activation of the NHEJ DNA repair pathway, we investigated the functional importance of MILIP in DSB DNA repair. MILIP knockdown triggered the appearance of comet tails and the formation of phosphorylated histone H2AX (γH2A.X) foci, phenocopying knockdown of Ku70 or Ku80, and treatment with the DNA-damaging agent etoposide (Fig. 4 *A*–*D* and *SI Appendix,* Fig. S5 *A*–*D*). Moreover, MILIP knockdown caused the accumulation of p53-binding protein 1 that was colocalized with γH2A.X (*SI Appendix,* Fig. S5*E*). Together, these results substantiate that MILIP plays a role in maintaining genomic integrity. In support, etoposide treatment resulted in increases in the colocalization of MILIP with γH2A.X and Ku70 similar to the increased recruitment of the Ku70 to DSB ends (*SI Appendix,* Fig. S5 *F*–*H*). Of note, p53 knockout did not significantly alter the formation of γH2A.X foci caused by knockdown of MILIP (*SI Appendix,* Fig. S5 *I* and *J*), suggesting that p53 is not involved in induction of DSB caused by knockdown of MILIP.

To test whether the association of MILIP with Ku70 and Ku80 is necessary for its effect on DSB DNA repair, we introduced an shRNA-resistant MILIP mutant (MILIP-sh.R) or a MILIP mutant with its -991/-1487 or -617/-990 fragment deleted, which could not bind to Ku70 or Ku80, into BE(2)-C.shMILIP and CHP-134.shMILIP cells. While introduction of MILIP-sh.R diminished the appearance of comet tails, formation of γH2A.X foci, and reduction in cell viability caused by MILIP knockdown (Fig. 4 *E*–*G* and *SI Appendix,* Fig. S5 *K* and *L*), introduction of the MILIP mutants did not affect these events (Fig. 4 *E*–*G* and *SI Appendix,* Fig. S5 *K* and *L*). Importantly, establishing the effects of MILIP-sh.R was mediated through Ku70–Ku80; knockdown of either Ku70 or Ku80 diminished the restoration of cell viability caused by introduction of MILIP-sh.R into BE(2)-C.shMILIP and CHP-134.shMILIP cells following the knockdown of endogenous MILIP (*SI Appendix,* Fig. S6*A*). Furthermore, Ku70 or Ku80 knockdown abolished the enhanced cell proliferation caused by MILIP overexpression (*SI Appendix,* Fig. S6 *B* and *C*). Therefore, binding to Ku70 and Ku80 is essential for MILIP to promote DSB DNA repair and cell survival and proliferation in neuroblastoma.

To corroborate that MILIP promotes NHEJ signaling, we employed DSB repair GFP reporter assays (15, 26). Notably, BE(2)-C and CHP-134 cells with MILIP knockdown exhibited a significant reduction in the number of GFP-positive cells in the NHEJ reporter assay, but the number of GFP-positive cells marking HR did not alter in cells with or without MILIP knockdown (Fig. 4*H*). Consistently, along with Ku70 and Ku80, other key components of the NHEJ complex, namely DNA ligase 4 and XRCC4, were recovered with MILIP in neuroblastoma cells (*SI Appendix*, Fig. S6*D*). Together, these results substantiate the role of MILIP in promoting NHEJ in neuroblastoma cells.

N-Myc is known to recruit the nuclear exosome complex to its target promoters to resolve TRCs, thus preventing the accumulation of DSBs (3). As we found that MILIP is transcriptionally activated by N-Myc (Fig. 1 *C*–*E* and *SI Appendix*, Fig. S1 *E*–*H*), we tested whether MILIP affects the recruitment of EXOSC10, one of the catalytic subunits of the nuclear RNA exosome, to the core promoters of the N-Myc-regulated genes, *NPM1* and *CDC7* (3). As anticipated, knockdown of N-Myc reduced the amount of EXOSC10 recovered with the *NPM1* and *CDC7* promoters (*SI Appendix,* Fig. S6*E*) (3). However, MILIP knockdown did not change the amount of EXOSC10 recruited to the promoters (*SI Appendix,* Fig. S6*E*). Therefore, MILIP knockdown-induced DNA DSBs are not closely associated with unresolved TRCs in neuroblastoma cells.

### MILIP Protects Neuroblastoma from DNA-Damaging Therapeutics.

**Fig. 5.**
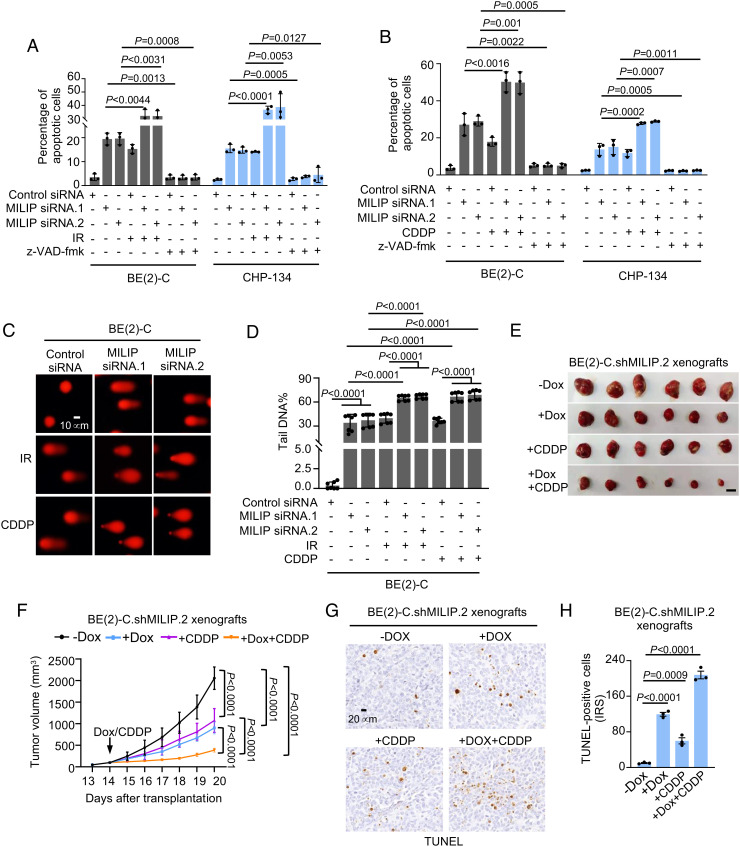
MILIP protects against DNA-damaging therapeutics. (*A* and *B*) MILIP knockdown and IR (*A*) or CDDP (*B*) cooperatively induced apoptosis. (*C*) MILIP nockdown and IR (*A*) or CDDP (*B*) cooperatively induced comet tails. (*D*) Quantitation of the relative tail DNA content as shown in *C*. (*E* and *F*) Photographs (*E*) and growth curves (*F*) of BE(2)-C.shMILIP.2 xenografts in nu/nu mice with or without treatment with Dox, CDDP, or Dox plus CDDP (n = 6 mice per group). (Scale bar, 1 cm.) (*G*) Representative microscopic photographs of TUNEL staining on randomly selected BE(2)-C.shMILIP.2 tumors (n = 3 tumors). (*H*) Quantitation of TUNEL staining as shown in *G* (n = 3 tumors). IRS: immunoreactive score. Data shown are mean ± SEM (*A*, *B*, and *D*) or representative (*C*) of 3 independent experiments. One-way ANOVA followed by Tukey’s multiple comparison test.

We investigated the potential of MILIP in regulating neuroblastoma cell sensitivity to ionizing radiation (IR) and cisplatin (CDDP), which are commonly used for neuroblastoma treatment and known to exert therapeutic effects mainly through DSBs (27). MILIP knockdown and IR or CDDP cooperatively induced apoptosis in BE(2)-C and CHP-134 cells (Fig. 5 *A* and *B*), increasing formation of comet tails and γH2A.X foci (Fig. 5 *C* and *D* and *SI Appendix,* Fig. S7 *A*–*D*). In contrast, MILIP overexpression protected, although moderately, SK-N-FI and SK-N-AS cells from CDDP-induced inhibition of cell viability (*SI Appendix,* Fig. S7*E*).

We also treated nu/nu mice carrying established BE(2)-C-shMILIP xenografts with Dox (to induce knockdown of MILIP), CDDP, or Dox plus CDDP. Cotreatment with Dox and CDDP inhibited BE(2)-C.shMILIP xenograft growth to a markedly greater extent compared with treatment with Dox or CDDP alone (Fig. 5 *E* and *F* and *SI Appendix,* Fig. S7*F*). This was associated with increased apoptosis (Fig. 5 *G* and *H*). Thus, MILIP expression contributes to neuroblastoma resistance to DNA-damaging therapeutics.

### Therapeutically Targeting MILIP Inhibits Neuroblastoma Xenograft Growth.

**Fig. 6.**
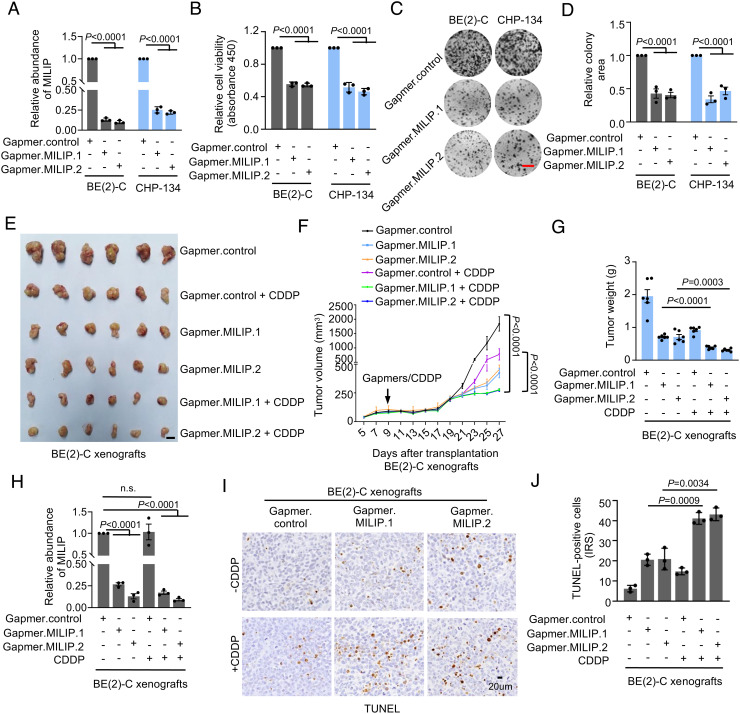
MILIP Gapmers inhibit, and in cooperation with CDDP to inhibit, neuroblastoma xenograft growth. (*A*) MILIP expression in cells with or without transfection of Gapmer.MILIP. (*B* and *C*) Introduction of Gapmer.MILIP reduced cell viability (*B*) and clonogenicity (*C*). (Scale bar, 1 cm.) (*D*) Quantitation of relative clonogenicity as shown in *C*. (*E* and *F*) Photographs (*E*) and growth curves (*F*) of BE(2)-C xenografts in nu/nu mice treated as indicated (n = 6 mice per group). (Scale bar, 1 cm.) (*G*) Quantitation of tumor weights as shown in *E* and *F* showing that cotreatment with Gapmer.MILIP (10 mg/kg, i.v. injection) and CDDP (1 mg/kg, i.p. injection) induced greater inhibition of BE(2)-C tumor growth than treatment with Gapmer.MILIP or CDDP alone in nu/nu mice (n = 6 mice per group, mean ± SEM, one-way ANOVA followed by Tukey’s multiple comparison test). (*H*) MILIP expression in representative BE(2)-C tumors (n = 3 tumors per group). (*I*) Representative microscopic photographs of TUNEL staining on randomly selected tumor tissues from mice treated in *E* and *F* (n = 3 tumors per group). (*J*) Quantitation of TUNEL staining as shown in *I* (n = 3 tumors per group). IRS: immunoreactive score. Data shown are mean ± SEM (*A*, *B*, *D*, and *G*) or representative (*C*) of 3 independent experiments. One-way ANOVA followed by Tukey’s multiple comparison test.

To further examine the therapeutic potential of MILIP targeting in the treatment of neuroblastoma, we employed Gapmers against MILIP (15, 28). Similar to siRNA and shRNA knockdown (Fig. 2 *A*–*I* and *SI Appendix,* Fig. S3 *A* and *E*), introduction of Gapmer.MILIP.1 and Gapmer.MILIP.2 diminished MILIP expression, inhibited cell viability, and reduced clonogenicity in BE(2)-C and CHP-134 cells (Fig. 6 *A*–*D*). Cotransfection of MILIP mutants carrying mismatches in the targeting sequences rescued the cells (*SI Appendix,* Fig. S8 *A* and *B*), validating the specificity of the Gapmers and further consolidating the role of MILIP in promoting neuroblastoma cell survival and proliferation.

We then treated nu/nu mice carrying established BE(2)-C or CHP-134 xenografts with Gapmer.MILIP.1 or Gapmer.MILIP.2 alone or in combination with CDDP. Treatment with Gapmer.MILIP retarded the growth of BE(2)-C and CHP-134 xenografts (Fig. 6 *E*–*H* and *SI Appendix,* Fig. S8 *C–F*). Moreover, the combination of Gapmer.MILIP.1 or Gapmer.MILIP.2 and CDDP markedly enhanced the inhibitory effect on tumor growth (Fig. 6 *E*–*H* and *SI Appendix,* Fig. S8 *C–F*), which was associated with increased apoptosis (Fig. 6 *I* and *J* and *SI Appendix,* Fig. S8 *G* and *H*). Importantly, treatment with Gapmer.MILIP did not cause any notable adverse reactions or weight loss (*SI Appendix,* Fig. S8 *I* and *J*), indicative of good tolerability of MILIP targeting in vivo.

## Discussion

Although N-Myc has long been postulated to regulate DNA DSB repair in neuroblastoma cells (4, 29), experimental evidence in support of this role of N-Myc has been sparse (29, 30). In this study, we demonstrated that the N-Myc-responsive lncRNA MILIP functions as an RNA scaffold that facilitates Ku70–Ku80 heterodimerization to promote the NHEJ pathway, thus substantiating the role of N-Myc in regulating DNA DSB repair in neuroblastoma cells (*SI Appendix,* Fig. S9).

Several lncRNAs have been documented to contribute to DNA DSB repair (14–16), but MILIP is distinguished by its role in the formation of the Ku complex essential for executing the first step of the NHEJ pathway (25, 31, 32). Therefore, as the transcription factor that drives MILIP expression, N-Myc appears to have a unique role in the activation of NHEJ in neuroblastoma cells. This not only promotes survival and proliferation of neuroblastoma cells undergoing genotoxic stress but may also be involved in their genomic instability, given the error-prone feature of the NHEJ mechanism (5, 33). Of note, while MILIP acts in the cytoplasm to repress p53 expression in some other cancer types (19), its effect on neuroblastoma cells takes place in the nucleus and is largely independent of p53, in agreement with the notion that lncRNAs often function in a highly tissue- and cell type–specific manner (13). As N-Myc is known to transcriptionally activate p53 (34), it is conceivable that MILIP and p53 act in concert downstream of N-Myc, with MILIP protecting cells from the cytotoxic effect of p53 to ensure DNA DSB repair in neuroblastoma cells (26, 27). Supporting this perception, neuroblastoma cells were arrested at the G1/G0 phase or were killed via apoptosis after MILIP silencing, both of which are typical manifestations of the p53 response to DNA damage (19, 27).

The Ku complex recognizes and binds to DNA DSB ends, which then functions as a scaffold to recruit a large battery of proteins involved in NHEJ DNA repair (8, 35, 36). Our results now reveal that MILIP is important for the interaction between Ku70 and Ku80 to form the Ku complex, functioning as an RNA platform to support “the Ku scaffold.” This was shown by the following: 1) MILIP knockdown diminished the association between Ku70 and Ku80, 2) Ku70 and Ku80 formed a ternary structure with MILIP, and 3) distinct structural regions in MILIP support binding to Ku70 and Ku80. Moreover, the functional significance of MILIP as a platform supporting the Ku70–Ku80 interaction was evident by the finding that interrupting the binding of MILIP to Ku70 or Ku80 diminished its protective effect on genomic integrity. Intriguingly, Ku70 and Ku80 are highly abundant proteins with approximately 500,000 molecules per cell (36), whereas MILIP, like many other lncRNAs (19, 26, 37), is expressed at noticeably lower abundance, with approximately 128–286 molecules per neuroblastoma cell. Thus, a relevant question is whether the stoichiometric disparity in MILIP could be sufficient to impact the activation of NHEJ. It is known that Ku proteins do not accrue at DSB ends in large numbers, with only one or two Ku molecules recruited to a DNA DSB end (35, 36, 38). It is therefore likely that the actual difference between the number of MILIP and Ku molecules is not as large as estimated at face value. Nevertheless, how MILIP distinguishes between Ku70 and Ku80 molecules in the general pool and those recruited to DSBs remains to be clarified. Regardless, that MILIP facilitates the Ku70–Ku80 association is not in dispute and its action appears sufficient to activate the NHEJ pathway in neuroblastoma cells.

Whether Ku70 and Ku80 can exist independently of the Ku complex as monomers remains unclear (35, 36). Ku70-deficient cells display low levels of Ku80, whereas K80-deficient cells also expressed low levels of K70 (39, 40). This is thought to arise due to the instability of Ku70 and Ku80 in the absence of their binding partners (39, 40). However, while MILIP knockdown reduced the Ku70–Ku80 interaction, it did not affect Ku70 and Ku80 expression, suggesting that dimerization may not be an absolute requirement for K70 and Ku80 expression in neuroblastoma cells. Both Ku70 and Ku80 proteins can be posttranslationally modified by diverse mechanisms, such as phosphorylation and acetylation, which are often involved in regulation of protein stability (41–44). Whether these modifications occur in a context-dependent manner to regulate Ku70 and Ku80 stability in neuroblastoma cells remains to be clarified. Similarly, whether the MILIP interaction with Ku70 or Ku80 is subjected to regulation by these modifications needs further investigation.

A practical implication of this study involves possible applications in the management of neuroblastoma. MILIP expression is frequently increased in neuroblastoma and is potentially an independent prognostic factor for patients. Our results clearly demonstrate that MILIP promotes neuroblastoma development and progression. Targeting the Ku70 and Ku80 proteins to disrupt their association has been proposed for cancer treatment (45, 46), with our results suggesting that MILIP represents an alternative yet potent avenue for inhibiting the Ku70–Ku80 interaction. Practically, this can be achieved using the Gapmer technology (28, 47), or alternatively, small molecules could be identified that block the interaction of MILIP with Ku70 and Ku80 (48, 49). These approaches will be of great interest toward future applications in neuroblastoma treatment.

## Materials and Methods

The information on human cell lines used is provided in *SI Appendix*, Table S3 and described in the extended supporting information along with the information about human tissues. Detailed experimental methods about cell viability, apoptosis, cell cycle, colony formation, immunoprecipitation, subcellular fractionation, immunofluorescence, ISH, IHC, comet assays, siRNA, inducible shRNA, CRISPR/Cas9 knockout of p53, quantitative PCR, luciferase reporter assays, DSB repair reporter assays, absolute quantification of RNA, western blotting, chromatin immunoprecipitation, RPD, RNA immunoprecipitation, mass spectrometry, in vitro transcription and xenograft mouse models, statistical analysis approaches, and data availability statement are also provided in the extended supporting information. Abs and reagents used are detailed in *SI Appendix*, Tables S4 and S5, respectively. Primer, probe, siRNA, and shRNA sequences are listed in *SI Appendix*, Tables S6 and S7. Treatment protocols of tumor-bearing mice are provided in *SI Appendix*, Table S8.

## Supplementary Material

Appendix 01 (PDF)

## Data Availability

The mass spectrometry proteomics data have been deposited in ProteomeXchange Consortium (PXD033372). All study data are included in the article and/or *SI Appendix* .
