# Supplementary material for: The N-Myc-responsive lncRNA MILIP promotes DNA double-strand break repair through non-homologous end joining
Source: Proc Natl Acad Sci U S A. 2022 Nov 29;119(49):e2208904119. doi: 10.1073/pnas.2208904119 (PMC9894261; doi:10.1073/pnas.2208904119)
Supplement: Supplementary file 1 — Appendix 01 (PDF) [file pnas.2208904119.sapp.pdf]

PNAS

Supporting Information for

The N-Myc-responsive lncRNA MILIP promotes DNA double-strand break repair through non-homologous end joining

Pei Lin Wang,[1,8] Liu Teng,[1,8] Yu Chen Feng,[2] Yi Meng Yue,[1] Man Man Han,[1] Qianqian Yan,[1] Kaihong Ye,[1] Cai Xia Tang,[1] Sheng Nan Zhang,[1] Teng Fei Qi,[1] Xiao Hong Zhao,[3] Ting La,[3] Yuan Yuan Zhang,[3] Jin Ming Li,[1] Bin Hu,[4] Dengfei Xu,[4] Shundong Cang,[4] Li Wang,[1,5] Lei Jin,[1,2] Rick F. Thorne,[1,3] Yuwei Zhang,[6]* Tao Liu,[1,7]* Xu Dong Zhang[1,3]*

Xu Dong Zhang
Email: Xu.Zhang@newcastle.edu.au

This PDF file includes:

Supporting text
Figures S1 to S9
Tables S1 to S8

Supporting Information Text

Materials and Methods.

Cell culture and human tissues

The information on human cell lines used is provided in Table S3. All cell lines were verified to be free of *Mycoplasma sp.* contamination every 3 months using RT-PCR. Individual cell line authentication was confirmed using the AmpFISTR Identifiler PCR Amplification Kit (ThermoFisher Scientific, 4427368) and Gene-Marker V1.91 software (SoftGenetics LLC).

Formalin-fixed paraffin-embedded (FFPE) human neuroblastoma and normal peripheral nerve tissue microarrays were purchased from the Servicebio (Wuhan, China, YNB642c). Studies using human tissues were approved by the Human Research Ethics Committees of the Henan Provincial People's Hospital in agreement with the guidelines set forth by the Declaration of Helsinki.

Cell viability

Cells were seeded at 4 × 10^3/well in 96-well plates overnight before treatment. Cell Counting Kit-8 (CCK8) solution (Med Chem Express, HY-K0301) was added at 37 °C for 2 h and the absorbance at 450 nm was recorded using a Varioskan LUX microplate reader (ThermoFisher Scientific).

Apoptosis

Apoptotic cells were quantitated using the Annexin V Apoptosis Detection Kit (BD Biosciences, 556547) according to manufacturer's instructions. Briefly, cells were resuspended in binding buffer and incubated with Annexin V/propidium iodide (PI) for 15 min at room temperature in dark before analysis using a flow cytometer (FACSCanto II, BD Biosciences).

Cell cycle analysis

Cells were fixed by 70% ethanol on ice for 1 h and spun down at 1500 × g. Cell pellets were re-suspended in PBS containing 0.25% Triton X-100 and incubate on ice for 15 min. After discarding the supernatant, Cell pellet was resuspended in 0.5 ml PBS containing 10 μg/mL RNase A and 20 μg/mL PI stock solution and incubate at room temperature (RT) in the dark for 30 min. Cells were analyzed using a flow cytometer (FACSCanto, BD Biosciences).

Colony formation

Cells seeded in six-well plates (1.5 × 10^3/well) were allowed to grow for two weeks before fixation with methanol followed by staining with 0.5% crystal violet. The images were captured with a Bio-Rad GelDoc™ XR +imaging system (Bio-Rad). Colonies were quantified using ImageJ-plugin "ColonyArea".

Immunoprecipitation (IP)

Cells were lysed with lysis buffer (20mM Tris-HCl pH 8.6, 100mM NaCl, 20mM KCl, 1.5mM $MgCl_2$, 1% Triton X-100, complete™ EDTA-free Protease Inhibitor Cocktail) on ice for 1 h. Samples were subjected to centrifugation at 16,000 × g for 30 min. Supernatants were pre-cleaned by incubation with protein A/G agarose beads (Life Technologies, 20421) in the absence of the capture antibody. The pre-cleaned supernatants were then incubated with the specified antibodies at 4 °C overnight. Protein–antibody complexes were then captured using protein A/G agarose beads at 4 °C for 2 h mixing by rotation. The beads were then rinsed and boiled with washing buffer (25mM Tris,150mM NaCl, pH 7.2). The precipitates analyzed by immunoblotting. Alternatively for two-step IP experiments, whole cell lysates derived similarly were first immunoprecipitated with anti-Ku80 Ab before elution with Ku80 peptides. For the second IP, the eluents were incubated with control IgG

or anti-Ku70 Ab, followed by immunoblotting and RT-PCR analysis. Ten percent of the precipitates were reserved for analysis by immunoblotting and RT-PCR as input controls. All processes were under RNase free conditions.

Subcellular fractionation

Cells were incubated with hypotonic buffer A (10 mM Hepes pH 7.9, 10 mM KCl, 0.1 mM EDTA, 0.1 mM EGTA, 1 mM DTT, 0.15% Triton X-100, complete™, EDTA-free Protease Inhibitor Cocktail) and swollen on ice for 15 min. Samples were centrifuged for 3 min at 12,000 × g, and the supernatant collected as the cytoplasmic fraction. The pellets were rinsed once with cold PBS and nuclear proteins extracted using an equal volume of buffer B (20 mM Hepes pH 7.9, 400 mM NaCl, 1 mM EDTA, 1 mM EGTA, 1 mM DTT, 0.5% Triton X-100, complete™, EDTA-free Protease Inhibitor Cocktail) on ice for 15 min. Cytoplasmic and nuclear fractions were centrifuged at 16,000 × g for 20 min to remove insoluble debris.

Immunofluorescence (IF)

Cells grown on coverslips were fixed for 10 min (4% formaldehyde) at room temperature, washed using PBS and then permeabilized using permeabilization buffer (0.1% Triton X-100 in PBS containing 10% BSA) before incubation overnight at 4°C with primary Ab. After washing with ice cold PBS three times, cells were incubated with Alexa Fluor 488-conjugated secondary Ab or CY3-conjugated secondary Ab in the dark. After washing with permeabilization buffer, the coverslip was mounted using the Pro-Long™ Glass Antifade Mountant with NucBlue reagent (ThermoFisher Scientific, P36981). Photomicrographs were collected using epifluorescence microscopy (Leica SP8).

In situ hybridization (ISH)

ISH assays were performed using the RNAscope® 2.5 HD Detection Reagent-BROWN (Advanced Cell Diagnostics, #322310) according to the manufacturer's instructions. Briefly, FFPE tissue microarrays were deparaffinized in xylene for 5 min at RT twice, followed by dehybridization in 100% alcohol. After being air-dried, the tissue sections were incubated with hydrogen peroxide for 10 min at RT and washed in the distilled water five times. Then the sections were heated in target retrieval reagent to 100 °C for 20 min, followed by treatment with proteinase K and incubated in hybridization buffer. containing probes at 40 °C for 3 h. After being washed, the sections were incubated with 3,3′-diaminobenzidine (DAB), and counterstaining was carried out using hematoxylin. The percentage of positive cells was ranged from 0 to 100%. The intensity of staining (intensity score) was judged on an arbitrary scale of 0–4: no staining (0), weakly positive staining (1), moderately positive staining (2), strongly positive staining (3) and very strong positive staining (4). A reactive score (RS) was derived by multiplying the percentage of positive cells with staining intensity divided by 10. Two researchers who were blinded to the information of FFPE tissue microarrays examined the ISH slides independently. The RS of each tissue was presented as the average of scores derived by the two researchers.

Immunohistochemistry (IHC)

FFPE tissue microarrays were deparaffinized in xylene for 5 min at RT twice, followed by dehybridization in 100% alcohol. Antigen retrieval was performed in a pressure cooker for 20 min in 10 mM Tris with 1 mM EDTA (pH 9). Endogenous peroxidase activity was inhibited with 1.5% H_2O2 in methanol for 20 min followed by washing in PBS. Nonspecific binding was blocked using blocking buffer (PBS (pH 7.4), 3% serum, 1% BSA and 0.1% Tween) for 60 min at room temperature. Sections were then incubated with an anti-Ki67 antibody (Proteintech Group, 27309-1-AP, Wuhan, China) that were diluted in blocking buffer overnight at 4 °C. After washing twice with 0.1% PBS–Tween, slides were incubated with a secondary antibody (BOSTER Biological Technology, BM3894, Wuhan, China). After washing, sections were incubated with with 3,3′ -diaminobenzidine (DAB) (Sigma- Aldrich) followed by counterstaining with hematoxylin (Servicebio,

G1004, Wuhan, China). After dehydration, sections were mounted using Permount TM Mounting Medium (Servicebio, WG10004160, Wuhan, China). Slides were examined by two investigators. The percentage of positive cells was estimated from 0% to 100%. The intensity of staining (intensity score) was judged on an arbitrary scale of 0–4: no staining (0), weakly positive staining (1), moderately positive staining (2), strongly positive staining (3), and very strongly positive staining (4). An immunoreactive score (IRS) was derived by multiplying the percentage of positive cells with staining intensity divided by 10. Two researchers who were blinded to the information of FFPE tissue microarrays examined the IHC slides independently. The IRS of each tissue was presented as the average of scores derived by the two researchers.

Comet assays

The CometAssay Single Cell Gel Electrophoresis Assay Kit was used according to manufacturer's instructions (Trevigen, 4250-050-K). Briefly, 500 cells (1×10^5 cells/ml) were mixed with low-melting-point agarose on slides at 37 °C. After solidifying for 10 min at 4 °C, the slides were immersed in the lysis solution and then in freshly prepared alkaline unwinding solution to permit DNA unfolding followed by electrophoresis (21 V for 30 min). The slides were washed with double distilled H_2O twice, immersed in 75% ethanol for 5 min, stained with PI. The percentage of tail DNA content of the comet was measured with Comet Assay IV software (Perceptive Instruments).

SiRNA

SiRNA duplexes were obtained from GenePharma (Shanghai, China) and transfected using the Lipofectamine 3000 Transfection Kit (ThermoFisher Scientific, L3000-015). SiRNA sequences are provided in Table S7.

Inducible shRNA

ShRNA oligos purchased from TSINGKE Biological Technology (Beijing, China) were constructed into FH1-tUTG plasmid (a kind gift from A/Professor M. J. Herold, Walter and Eliza Hall Institute of Medical Research, Australia). The lentiviral particles were packaged via cotransfection with FH1-tUTG (44 µg), pMDLg.pRRE (22 µg), pMD2.g (13.2 µg), and pRSU.pREV (11 µg) plasmids into HEK293 cells in a T175 culture flask. Inducible knockdown cell sublines were established after lentiviral transduction. ShRNA sequences are provided in Table S7.

CRISPR/Cas9 knockout of p53

Single-guide RNA (sgRNA) sequences targeting p53 (5'-GGGCAGCTACGGTTTCCGTC-3') were cloned into the lentiCRISPR v2 plasmid (#52961, Addgene, Cambridge, MA, USA) by simple annealing and ligation. CHP-134 cells were transfected with the sgRNA construct using Lipofectamine 3000 reagent (ThermoFisher Scientific). Twenty-four hrs later, cells were subjected to selection in the culture medium containing puromycin (#A1113803, ThermoFisher Scientific) for 1 week. Viable cells were trypsinized, washed with the culture medium, and re-plated on 96 well plates in single cell suspension by limiting dilution and cultured for 12–14 days. Single cell colonies were picked, expanded, and assayed for p53 expression using Western blot analysis. The DAN indels in the TP53 locus of the two p53 knockout sublines (CHP-134.p53KO1 and CHP-134.p53KO2) were further confirmed by Sanger sequencing.

Quantitative PCR (qPCR)

Total RNA was extracted from cultured cells using the Gene JET RNA Purification Kit (ThermoFisher Scientific, #K0731) according to the manufacturer's instructions. cDNA was synthesized from 1 µg of total RNA using the PrimScriptTM RT reagent Kit with gDNA Eraser (TaKaRa, #RR047A; Dalian, China). Of the resultant cDNA, 12.5 ng was used in the 20 µl qPCR mix, containing 10 µl of TB Green Premix Ex Taq II (Tli RNaseH Plus) (TaKaRa, #RR820A; Dalian, China) and 0.4 µM of each primer. Samples were amplified for 40 cycles using a StepOnePlusTM

Real-Time PCR System (ThermoFisher Scientific). The 2-ΔΔCT method was used to calculate expression levels relative to the GAPDH or 18S rRNA housekeeping controls.

Luciferase reporter assays

The Dual-Luciferase® Reporter Assay System was performed according to manufacturer's instructions (Promega, E1910). Cells were transfected with the pGL3-based constructs containing MILIP promoter together with Renilla luciferase plasmids. Twenty-four hours later, Firefly and Renilla luciferase activities were examined by Varioskan LUX microplate reader (ThermoFisher). Renilla luciferase activities were used to normalize the firefly luciferase activity.

DSB repair reporter assays

The activity of DSB pathways was measured using GFP reporters for total NHEJ (EJ5-GFP; Addgene, 44026) and HR (DR-GFP; Addgene, 26475). Cells were co-transfected with MILIP or control siRNA, the reporter EJ5-GFP or DR-GFP, and a plasmid encoding the I-SceI endonuclease (Addgene, 26477) to introduce a DSB at I-SceI sites in the reporter constructs. GFP was measured using a flow cytometer (FACSCanto, BD Biosciences). A vector expressing GFP only was co-transfected in parallel to measure transfection efficiency. The percentage of GFP positive cells were normalized to transfection efficiency.

Absolute quantification of RNA

Absolute RNA quantification was performed using the standard curve method by qPCR. cDNA was prepared from a fixed cell number using the qScript cDNA SuperMix (Quantabio, Cat# 95048-500) in a 20 μL reaction and subsequently diluted to 100 μL. Ten-fold serial dilutions of the pcDNA3.1-MILIP plasmid (10^2–10^7 molecules per ml) were used as a reference molecule for the standard curve calculation. Assays were reconstituted to a final volume of 20 μl using 5 μl cDNA from cells or 5 μl serial diluted pcDNA3.1- MILIP plasmid and cycled using a StepOnePlusTM Real-Time PCR System. Data calculated as copies per 5 μl cDNA were converted to copies per cell based on the known input cell equivalents. The sequences for primers are provided in Table S6.

Western blotting

Cells were lysed in lysis buffer (50mM Tris-HCl [pH 7.5], 150mM NaCl, 2.5mM $MgCl_2$, 1mM EDTA, 10% Glycerol, 1% triton-100, 1mM DTT, complete™ EDTA-free protease inhibitor cocktail [Sigma-Aldrich, 4693132001]) and sonicated. Supernatant was collected after the samples were centrifugated at 13,000 × g at 4 °C for 20 min. Protein concentrations in the supernatant samples were quantified with the Bicinchoninic Acid Assay kit (Pierce, Rockford, IL). Equal amounts of protein samples were loaded onto each lane of sodium dodecyl sulfatepolyacrylamide (SDS) gel, followed by electrophoresis and transfer to nitrocellulose membranes. The membranes were blocked of nonspecific antibody binding with 10% skim milk powder in phosphate-buffered saline and probed with the primary antibody. After washing with TTBS, the membrane was then incubated with a horseradish peroxidase-conjugated goat anti-rabbit or goat anti-mouse antibody. Protein bands were visualized with SuperSignal West Pico Chemiluminescent Substrate (Pierce, 34079). Semi-quantitation of protein bands was carried out using the NIH ImageJ. The information of antibodies used is provided in Table S4.

Chromatin immunoprecipitation (ChIP)

ChIP assays were performed using the ChIP Assay Kit (Beyotime, #P2078; Shanghai, China) according to the manufacturer's instructions. Briefly, cells were cross-linked with a final concentration of 1% formaldehyde in growth medium for 15 min at 37 °C and quenched by the addition of glycine solution for 5 min at room temperature (RT). Then cells were harvested, lysed using cell lysis buffer (2% SDS [w/v], 150 mM NaCl, 50 mM Tris/HCl, 50 mM EDTA, pH 8.0) and sonicated. After being cleared by centrifugation at 12,000 × g for 10 min at 4 °C, the cell lysate was subjected to a 1:10 dilution and rotated with antibodies or corresponding mouse/rabbit normal

immunoglobulin at 4 °C overnight. Then, 60 µl of protein A/G agarose beads was added to the antibody-lysate mixture and rotated at 4 °C for an additional 1 h. Beads were washed using the lysis buffer, and DNA fragments were eluted, purified and subjected to PCR analysis using the specific primers. PCR products were separated by gel electrophoresis on the 2% agarose gel. The sequences for primers are provided in Table S6.

RNA pulldown (RPD)

Cell lysates were prepared with lysis buffer (50mM Tris-HCl [pH 7.5], 150mM NaCl, 2.5mM MgCl2, 1mM EDTA, 10% Glycerol, 1% triton-100, 1mM DTT, complete™ EDTA-free protease inhibitor cocktail [Sigma, 4693132001] and RiboLock RNase inhibitor [Life Technologies, EO0382]) followed by incubation with probes before rotating with streptavidin beads (ThermoFisher, 20349) for 2 hrs. The beads were washed in the lysis buffer five times and the retrieved proteins were separated by SDS/PAGE for mass spectrometry or immunoblotting. The sequences for probes are provided in Table S6.

RNA immunoprecipitation (RIP)

The EZ-Magna RIP Kit (Millipore, 17-701) was used according to manufacturer's instructions. Briefly, whole cell lysates prepared by ultrasonication in lysis buffer (50mM Tris-HCl [pH 7.5], 150mM NaCl, 2.5mM $MgCl_2$, 1mM EDTA, 10% Glycerol, 1% triton-100, 1mM DTT, complete™ EDTA-free protease inhibitor cocktail [Sigma, 4693132001] and RiboLock RNase inhibitor [Life Technologies, EO0382]) were incubated with magnetic beads coated with the indicated Abs at 4 °C. After washing with lysis buffer 5 times, the bead-bound immunocomplexes were treated with proteinase K. Samples were then centrifuged and placed on a magnetic separator. Supernatants were used to extract RNA with a FastPure® Cell/Tissue Total RNA Isolation Kit V2 (Vazyme, RC112-01) before subjecting the purified RNAs to PCR analysis. The sequences for primers are provided in Table S6.

Mass spectrometry (MS) analysis.

Cell lysates were prepared by ultrasonication in lysis buffer (50mM Tris-HCl [pH 7.5], 150mM NaCl, 2.5mM $MgCl_2$, 1mM EDTA, 10% Glycerol, 1% triton-100, 1mM DTT, complete™ EDTA-free protease inhibitor cocktail [Sigma, 4693132001] and RiboLock RNase inhibitor [Life Technologies, EO0382]) followed by incubation with probes before rotating with streptavidin beads (ThermoFisher, 20349) for 2 hrs. The beads were washed in the lysis buffer five times and the retrieved proteins were separated by 12.5% SDS PAGE. Protein bands were visualized by Coomassie blue staining. Two bands at ~70 kDa and ~80 kDa, respectively, were observed in samples derived with MILIP antisense probes but not in samples derived with MILIP sense probes. The segment of the SDS gel corresponding to each band was cut out and processed for MS analysis. Peptides were sequenced by nanoflow reversed phased Liquid Chromatography (Dionex Ultimate 3000 RSLCnano, Dionex, Idstein, Germany) coupled directly to an ESI 3D Ion Trap Mass Spectrometer (Luming Biotechnology, Shanghai, China) operating in MS/MS (CID) mode (n = 1 technical replicate). Peptides were loaded at 5 µl/min onto a 5 µm C18 nanoViper trap column (100 µm × 2 cm, Acclaim PepMap100, Thermo) for desalting and pre-concentration. Peptide separation was then performed at 300 nl/min over an Acclaim nanoViper analytical column (2 µm C18, 75 µm × 15 cm) utilising a gradient of 2–40% Buffer B (80% Acetonitrile, 0.1% Formic Acid) over 60 min. The peptides were eluted directly into the nanoflow ESI Ion source of the MS system for MS/MS analysis. The AmaZon Ion Trap system was tuned using Smart Parameter Settings tuned to 922 m/z and set to perform MS/MS on the top 6 ions present in each MS scan with an Ion exclusion time of 30 sec. Source settings were as follows: dry gas temperature, 180 °C; dry gas, 4.0 L min−1; nebulizer gas, 0.4 bar; electrospray voltage, 4500 V; high-voltage endplate offset, –200 V; capillary exit, 140 V; trap drive, 57.4; funnel 1 in 100 V, out 35 V, and funnel 2 in 12 V, out 3.3 V; MS/MS ICC target 500,000; maximum accumulation time, 50 ms. The sample was measured with the Ultrascan Scan Mode in polarity positive, scan range from m/z 100–3000, 3 MSn spectra averages. MS/MS spectra were triggered on ions higher than 50,000 in the Profile scan using

a fragmentation amplitude of 100%.

Raw MS Files were converted into MASCOT Generic Format using DataAnalysis 4.1 and imported into ProteinScape 2.1 platform (both Bruker, Bremen, Germany) for database searching. Searches were performed against the UniProt Swiss-Prot Human database (retrieved January 2017) using an in-house licensed MASCOT server (version 2.3.02, Matrix Science). The number of allowed trypsin missed cleavages set to 2. Deamidation of Asparagine and Glutamine, Oxidation of Methionine and Phosphorylation of Serine, Threonine and Tyrosine were set as variable modifications. The parent ion tolerance was set to 1.2 Da with fragment ion tolerance set to 0.7 Da. Peptide thresholds were set requiring False Positive Rate less than 0.05% with a low stringency MASCOT score greater than 35. Those spectra meeting these criteria were validated by manual inspection to ensure accurate y- and b-ion detection with overlapping sequence coverage.

In vitro transcription

The plasmid pcDNA3.1-MILIP was constructed by TSINGKE Biological Technology (Beijing, China). The plasmids were linearized by restriction enzyme BstBI (New England biolab, R0519S) and in vitro transcription was then performed using TranscriptAid T7 High Yield Transcription Kit (ThermoFisher Scientific, K0441) according to the manufacturer's instructions.

Xenograft mouse model

Cells (5×10^6) were subcutaneously injected into the dorsal flanks of 4-week-old female nu/nu mice (GemPharmatech, Shanghai, China) (6 mice per group), Treatment protocols are detailed in Table S8. At the end of experiments, mice were sacrificed, and tumors excised and measured. Studies on animals were approved by the Animal Research Ethics Committee of the Academy of Medical Science of Zhengzhou University. *In vivo* treatment protocols were detailed in Table S8.

Statistical Analysis

Analysis was carried out using GraphPad Prism to assess differences between experimental groups. Statistical differences were analyzed by two-tailed Student's t-test or One-way ANOVA test followed by Tukey's multiple comparisons. P values lower than 0.05 were considered statistically significant.

Data availability

The mass spectrometry proteomics data have been deposited to the ProteomeXchange Consortium (http://proteomecentral.proteomexchange.org) with the dataset identifier PXD033372. All other data supporting the findings of this study are available from the corresponding authors on request.

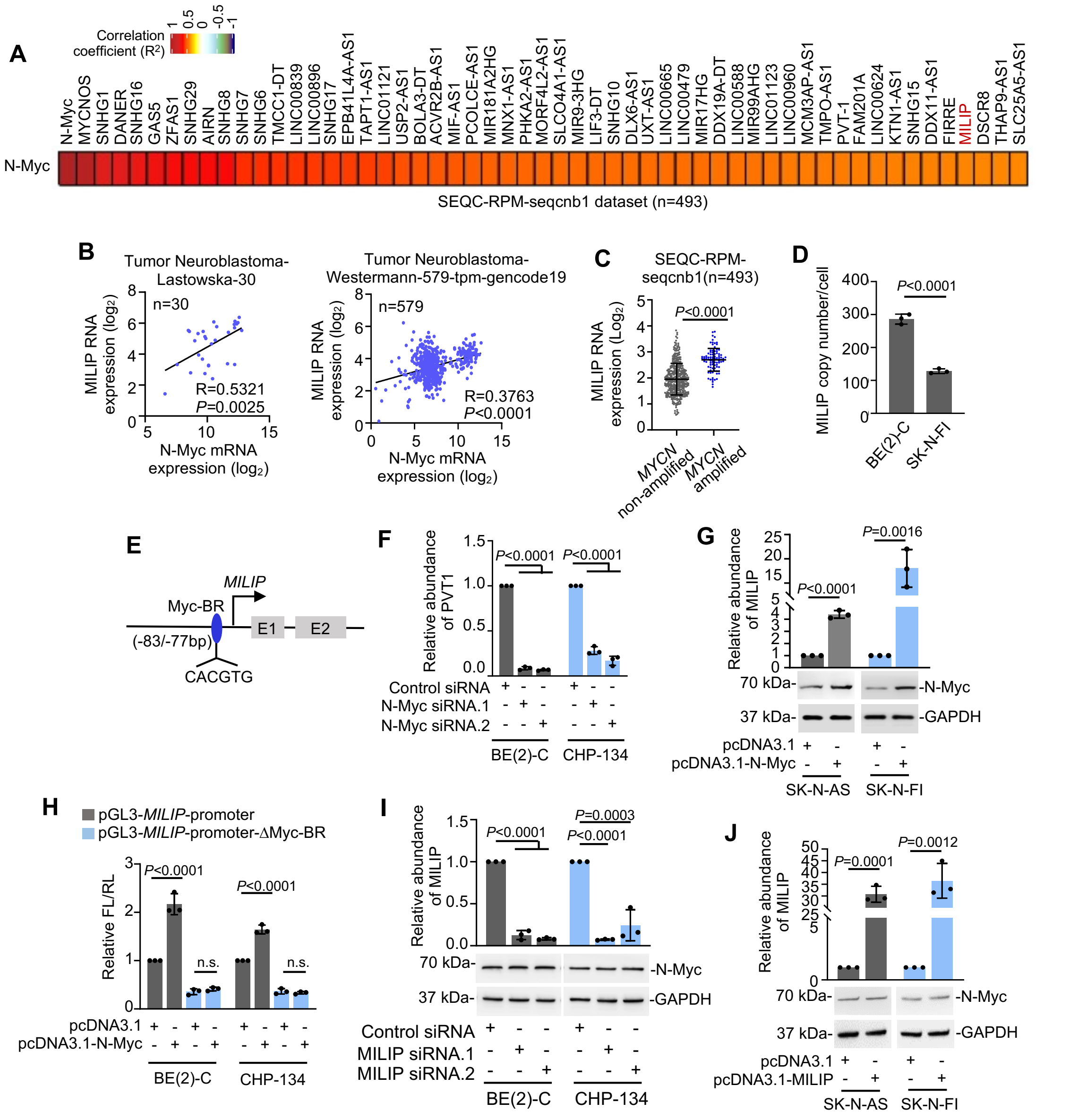

Fig. S1. N-Myc regulates MILIP expression in neuroblastoma. A, heatmap showing the correlation coefficient (R^2) generated using regression analysis between N-Myc mRNA expression and the expression of individual lncRNAs in the R2 RNA-seq SEQC-RPM-seqcnb1 neuroblastoma dataset. B, regression analysis showing a correlative relationship between MILIP and N-Myc mRNA expression in the Affymetrix microarray Lastowaska-30 dataset and the RNA-seq Westermann-579-tpm-gencode19 neuroblastoma datasets. The units of MILIP and N-Myc mRNA expression in the Lastowaska-30 dataset were obtained by analyzing hybridization signal intensity of individual probes (transcripts) on Affymetrix microarray slides with MAS5.0 algorism. The units of MILIP and N-Myc mRNA expression in the Westermann-579-tpm-gencode19 dataset are TPM. C, MILIP was expressed at higher levels in *MYCN*-amplified than *MYCN*-nonamplified neuroblastomas in the SEQC-RPM-seqcnb1 neuroblastoma dataset (two-tailed Student's *t*-test). D, absolute quantitation of MILIP showing that MILIP copy numbers were higher in each of *MYCN*-amplified (BE(2)-C) than *MYCN*-nonamplified (SK-N-FI) neuroblastoma cells (Data shown are mean ± SEM of 3 independent experiments. One-way ANOVA followed by Tukey's multiple comparison). E, schematic illustration of a consensus Myc binding region (Myc-BR) in *MILIP* promoter as identified

in ENCODE (Encyclopedia of DNA Elements). F, siRNA knockdown of N-Myc (as shown in Figure 1D) downregulated the expression of the lncRNA PVT1 (Data shown are mean ± SEM of 3 independent experiments. One-way ANOVA followed by Tukey's multiple comparison). G, N-Myc overexpression upregulated MILIP in SK-N-FI and SK-N-AS cells (Data shown are mean ± SEM of 3 independent experiments. Two-tailed Student's *t*-test). H, the transcriptional activity of a MILIP reporter construct containing the Myc-BR was increased by overexpression of N-Myc, whereas a MILIP reporter construct with the Myc-BR deleted displayed decreased activity that was not further affected by N-Myc overexpression (Data shown are mean ± SEM of 3 independent experiments. One-way ANOVA followed by Tukey's multiple comparison). I and J, neither knockdown (I) nor overexpression (J) of MILIP altered the expression of N-Myc as measured using immunoblotting (Data shown are mean ± SEM of 3 independent experiments. One-way ANOVA followed by Tukey's multiple comparison (I) or two-tailed Student's *t*-test (J).

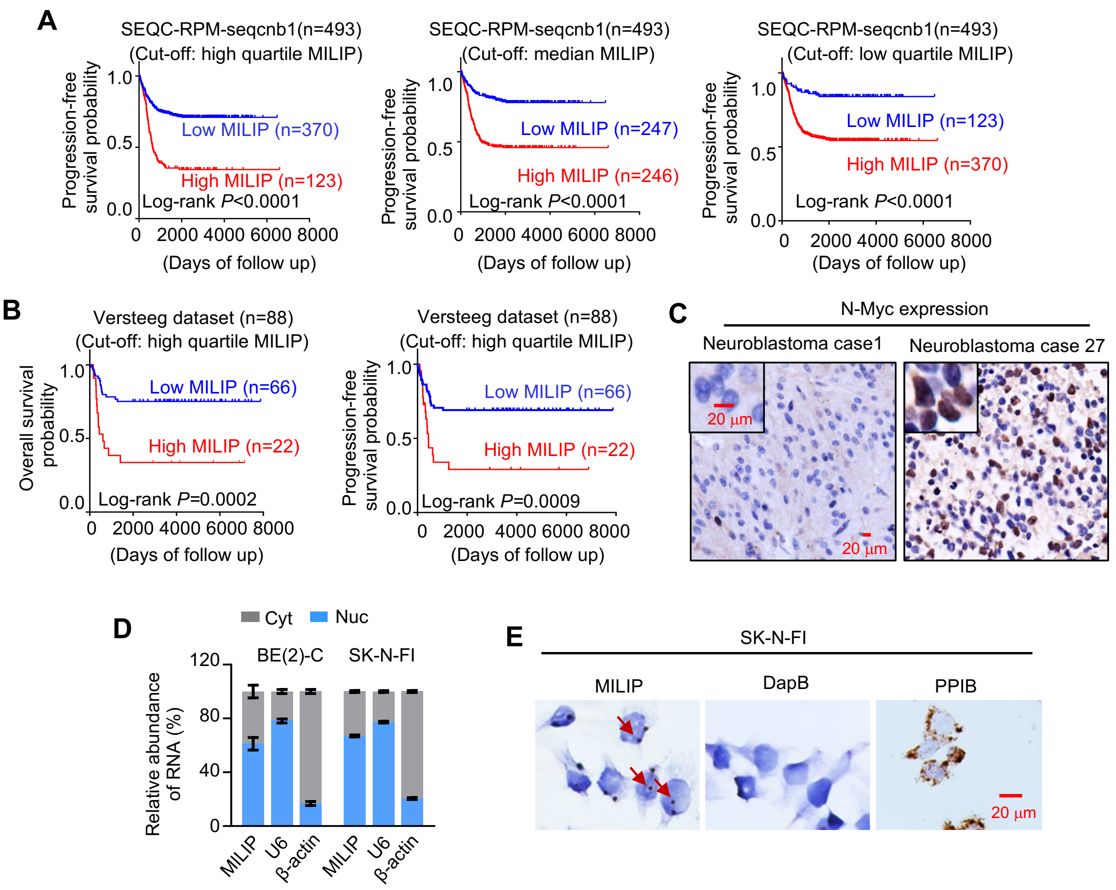

Fig. S2. High MILIP expression is associated with poor outcome of neuroblastoma patients. A, Kaplan–Meier analysis of the probability of progression-free survival of the 493 neuroblastoma patients in the SEQC-RPM-seqcnb1 dataset using the high quartile, median, and low quartile of MILIP expression levels as the cut off points. B, Kaplan–Meier analysis of the overall and probability of progression-free survival of the 88 neuroblastoma patients in the R2 Versteeg dataset using the high quartile of MILIP expression levels as the cutoff point. C, representative microscopic photographs of IHC staining of N-Myc in FFPE neuroblastoma tissue sections (n = 27). D, qPCR analysis of subcellular fractions of BE(2)-C and SK-N-FI cells showing a large proportion of MILIP was located to the nucleus. Cyt: cytoplasm; Nuc: nucleus. β-actin: Cytoplasmic marker; U6: Nuclear marker (Data shown are mean ± SEM of 3 independent experiments). E, representative microscopic photographs of in situ hybridization (ISH) analysis of MILIP expression in SK-N-FI cells grown on coverslips (Data shown are representative of 3 independent experiments). Staining of DapB and PPIB was included as negative and positive controls, respectively.

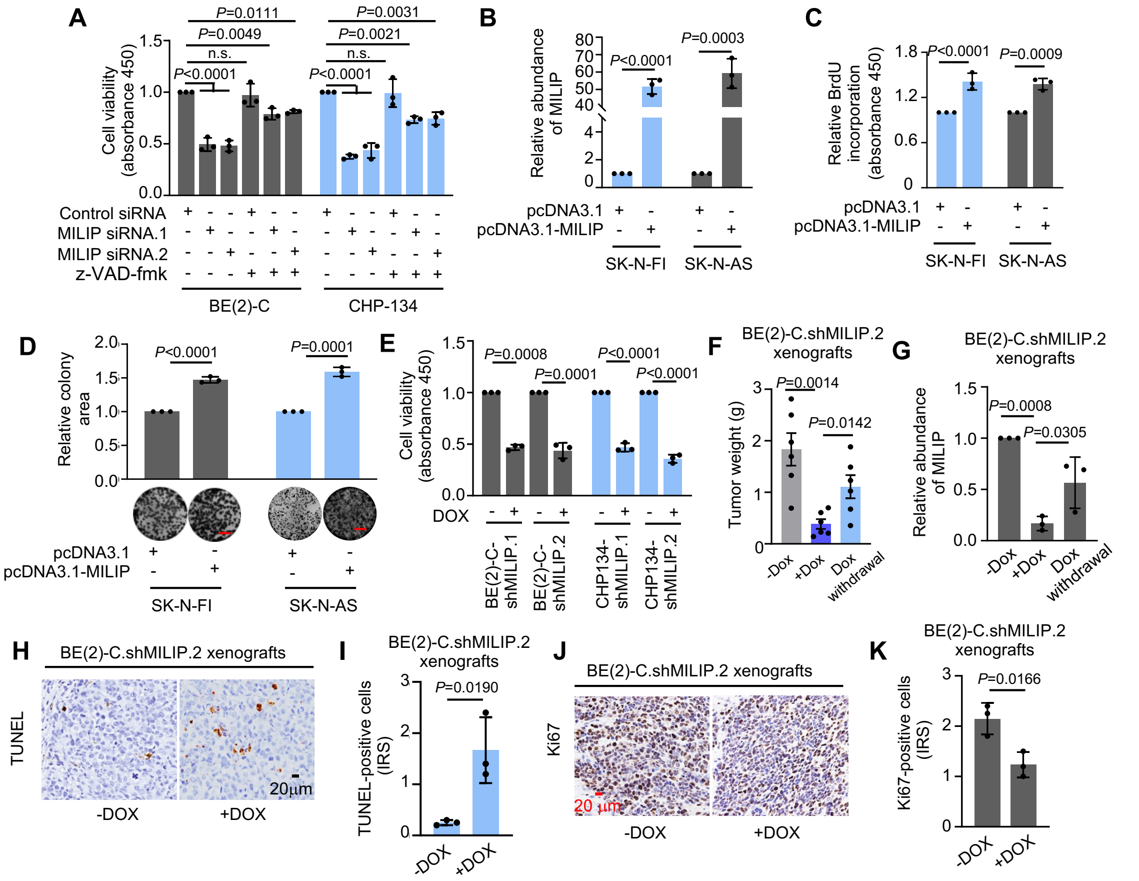

Fig. S3. MILIP promotes neuroblastoma cell survival, proliferation and tumorigenicity. A, the addition of z-VAD-fmk (20mM) partially inhibited the reduction in cell viability caused by MILIP knockdown in BE(2)-C and CHP-134 cells (Data shown are mean ± SEM of 3 independent experiments. One-way ANOVA followed by Tukey's multiple comparison). B, qPCR analysis showing that MILIP was overexpressed in SK-N-FI and SK-N-AS cells (Data shown are mean ± SEM of 3 independent experiments. Two-tailed Student's *t*-test). C, overexpression of MILIP promoted SK-N-FI and SK-N-AS cell proliferation as shown in BrdU incorporation assays (Data shown are mean ± SEM of 3 independent experiments. Two-tailed Student's *t*-test). D, overexpression of MILIP promoted SK-N-FI and SK-N-AS cell clonogenicity (Data shown are mean ± SEM (upper) or representative (lower) of 3 independent experiments. Two-tailed Student's t-test). Scale bar, 1 cm. E, induced knockdown of MILIP in BE(2)-C.shMILIP.1, BE(2)-C.shMILIP.2, CHP-134.shMILIP.1 and CHP-134.shMILIP.2 cells by the addition of doxycycline (Dox, 100 ng/mL) caused reductions in cell viability (Data shown are mean ± SEM of 3 independent experiments. Two-tailed Student's *t*-test).F, quantitation of tumor weights as shown in Figure 2J and 2K showing that treatment with Dox (2 mg/ml supplemented with 10 mg/ml sucrose in drinking water) caused reductions in BE(2)-C.shMILIP.2 xenograft wights in nu/nu mice that was however reversed by the cessation of Dox treatment (n = 6 mice per group, mean ± SEM, one-way ANOVA followed by Tukey's multiple comparison). G, qPCR analysis of randomly selected BE(2)-C.shMILIP.2 tumor tissues harvested from nu/nu mice with or without cessation of Dox treatment showing MILIP expression levels (n = 3 tumors). One-way ANOVA followed by Tukey's multiple comparison). H, representative microscopic photographs of TUNEL staining on FFPE tissue sections from representative BE(2)-C.shMILIP.2 xenografts harvested from mice with or without Dox treatment to induce knockdown of MILIP (n = 3 tumors per group). I, quantitation of TUNEL positive cells as shown in H (n = 3 tumors per group. Two-tailed Student's *t*-test). J, representative microscopic photographs of IHC staining of Ki67 on FFPE tissue sections from representative BE(2)-C.shMILIP.2 xenografts harvested from mice with or without Dox treatment to induce knockdown

of MILIP (n = 3 tumors per group). K, quantitation of Ki67 positive cells as shown in J (n = 3 tumors per group. Two-tailed Student's *t*-test). IRS: Immunoreactive score.

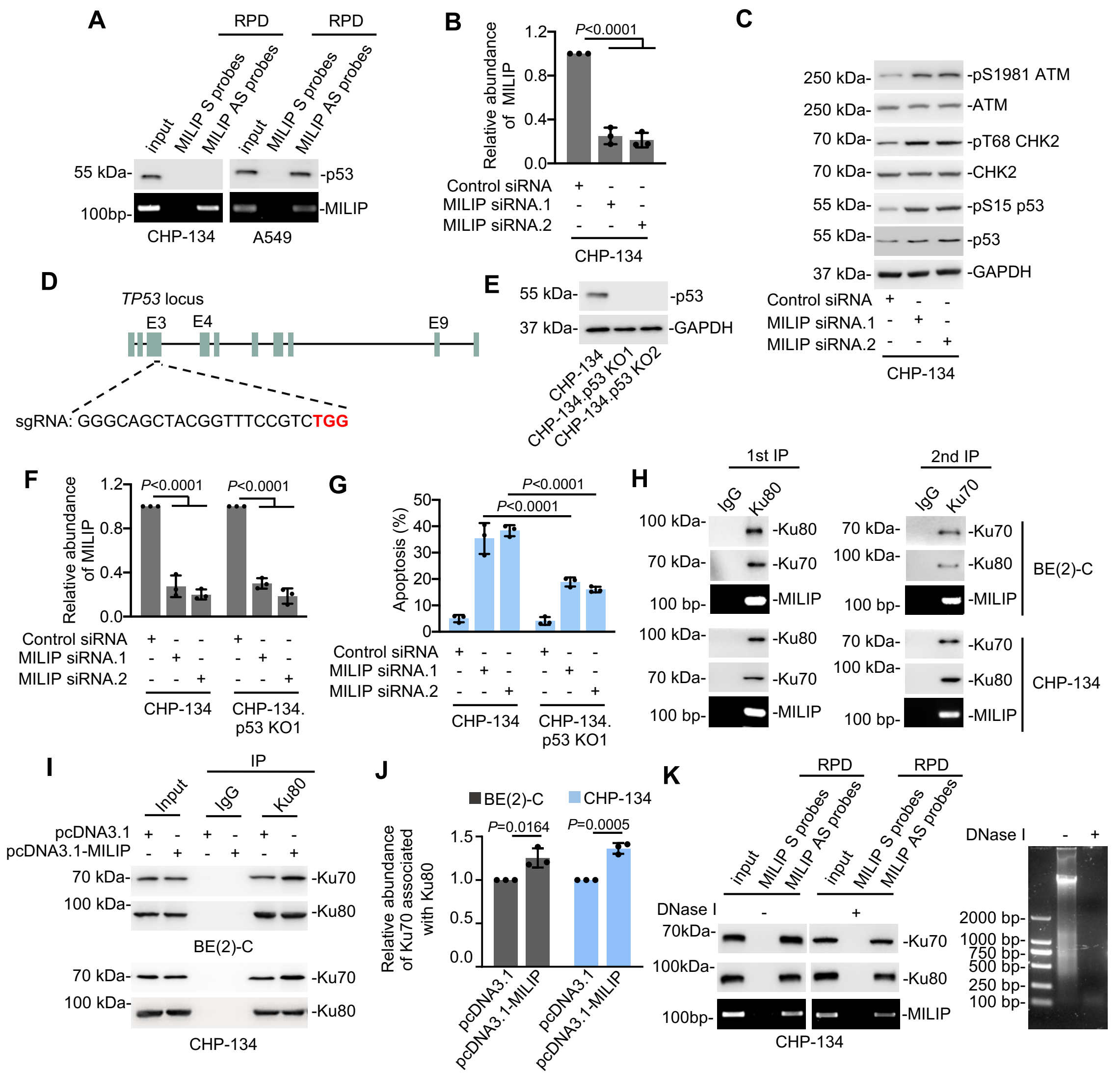

Fig. S4. MILIP binds to Ku70 and Ku80 and promotes their heterodimerization. A, p53 was co-pulled down in A549 but not in CHP-134 cells (Data shown are representative of 3 independent experiments). **B and C**, knockdown of MILIP (B) caused phosphorylation of ATM, CHK2 and p53 and moderate upregulation of p53 in CHP-134 cells (Data shown are representative of 3 independent experiments). **D**, schematic illustration of the targeting strategy to generate p53 knockout CHP-134 sublines (CHP-134.p53KO1 and CHP-134.p53KO2) using the CRISPR/Cas9 system. The proto-spacer adjacent motif (PAM) sequences are highlighted in red. **E,** Western blot analysis showing knockout of p53 in CHP-134.p53KO1 and CHP-134.p53KO2 cells (Data shown are representative of 3 independent Western blotting experiments). **F and G**, MILIP knockdown (F)-induced apoptosis was partially inhibited by p53 knockout as shown in CHP-134.p53KO1 cells (G) (Data shown are mean ± SEM of 3 independent experiments. One-way ANOVA followed by Tukey's multiple comparison). **H,** Ku70, Ku80 and MILIP were co-precipitated with an anti-Ku80 antibody in whole cell lysates of BE(2)-C and CHP-134 cells (left), and after elution, all were further co-precipitated with an anti-Ku70 antibody in the resultant precipitates (right) (Data shown are representative of 3 independent experiments). **I,** MILIP overexpression caused increases in the amount of Ku70 co-precipitated with Ku80 in BE(2)-C and CHP-134 cells (Data shown are representative of 3 independent experiments). **J,** quantitation of the relative amount of Ku70 associated with Ku80 in cells with or without MILIP overexpression as shown in **I**. Data shown are mean ± SEM of 3 independent experiments. Two-tailed Student's t-test). **K,** treatment of whole cell

lysates with DNase I (right) did not affect the interaction of MILIP with Ku70 and Ku80 as shown in RNA pulldown (RPD) assays (left) (Data shown are representative of 3 independent experiments).

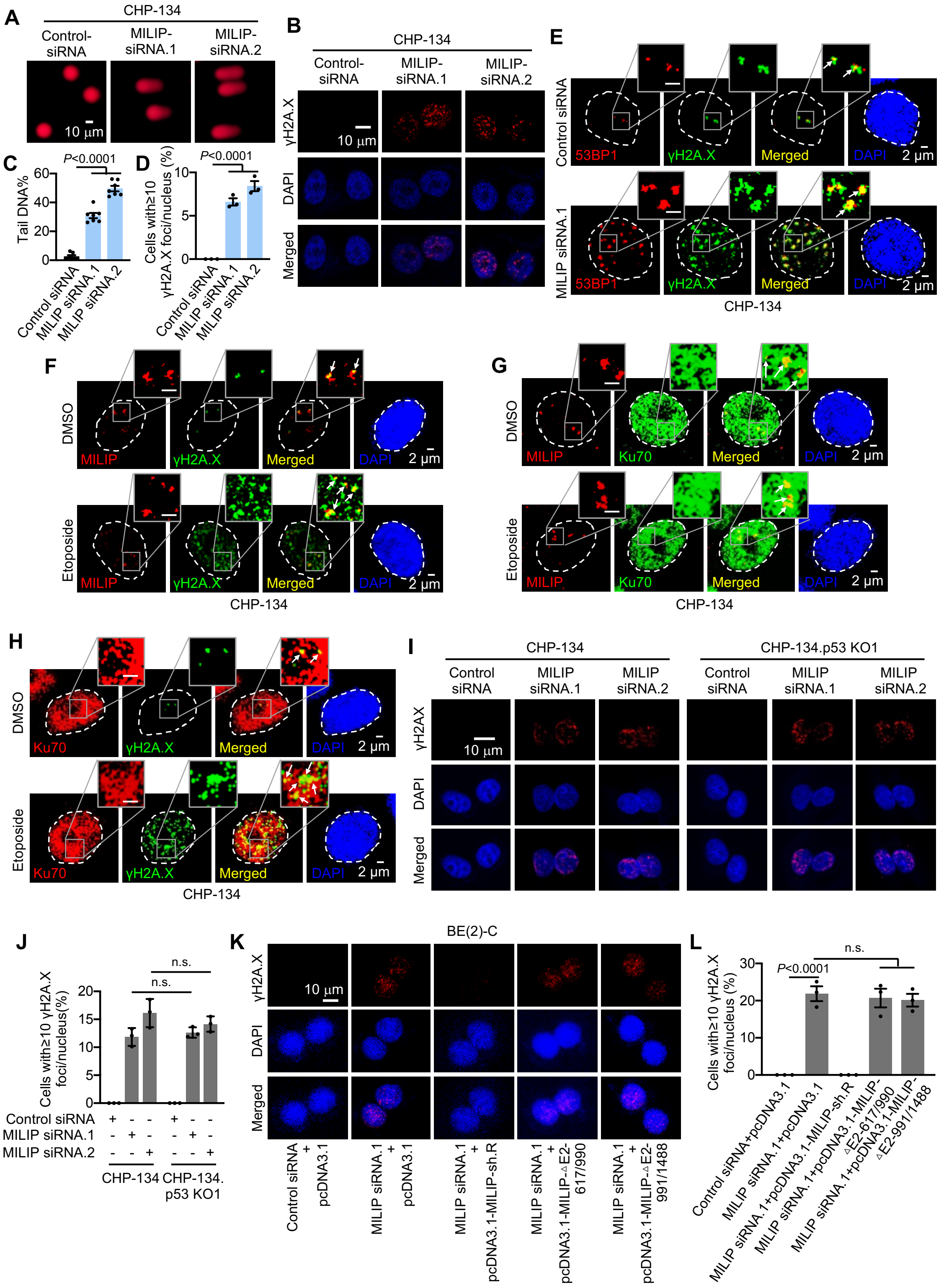

Fig. S5. MILIP promotes DNA DSB repair. A and B, MILIP knockdown induced the appearance of comet tails (A) and the formation of γH2A.X foci (red) (B) in CHP-134 cells (Data shown are representative of 7 (A) or 3 (B) independent experiments). **C and D**, quantitation of the relative tail DNA content of the comets as shown in A (C) and the percentage of cells with γH2A.X foci as shown in B (D) (Data shown are mean ± SEM of of 7 (C) or 3 (D) independent experiments. One-

way ANOVA followed by Tukey's multiple comparison). **E,** colocalization of 53BP1 and gH2A.X in CHP-134 cells with or without MILIP knockdown was identified using immunofluorescence staining of 53BP1 (red) and gH2A.X (green) (Data shown are representative of 3 independent experiments). **F,** colocalization of MILIP and gH2A.X in CHP-134 cells with or without etoposide treatment was identified using fluorescence in situ hybridization (FISH) analysis of MILIP (red) in conjunction with immunofluorescence staining of gH2A.X (green) in CHP-134 cells (Data shown are representative of 3 independent experiments). **G,** colocalization of MILIP and Ku70 was identified using FISH analysis of MILIP (red) in conjunction with immunofluorescence staining of Ku70 (green) in CHP-134 cells with or without etoposide treatment (Data shown are representative of 3 independent experiments). **H,** colocalization of Ku70 and gH2A.X in CHP-134 cells with or without treatment with etoposide was identified using immunofluorescence staining of Ku70 (red) and gH2A.X (green) (Data shown are representative of 3 independent experiments). **I,** MILIP knockdown caused the formation of γH2A.X foci (red) in CHP-134 cells and CHP-134.p53KO1 cells (Data shown are representative of 3 independent experiments). **J,** quantitation of the percentage of cells with γH2A.X foci as shown in I (Data shown are mean ± SEM of 3 independent experiments. One-way ANOVA followed by Tukey's multiple comparison). **K,** introduction of MILIP-sh.R but not MILIP-△-617/-990 or MILIP-△-991/-1487 diminished the formation of γH2A.X foci (red) caused by knockdown of endogenous MILIP in BE(2)-C cells (Data shown are representative of 3 independent experiments). **L,** quantitation of the percentage of cells with γH2A.X foci as shown in E (Data shown are mean ± SEM of 3 independent experiments. One-way ANOVA followed by Tukey's multiple comparison).

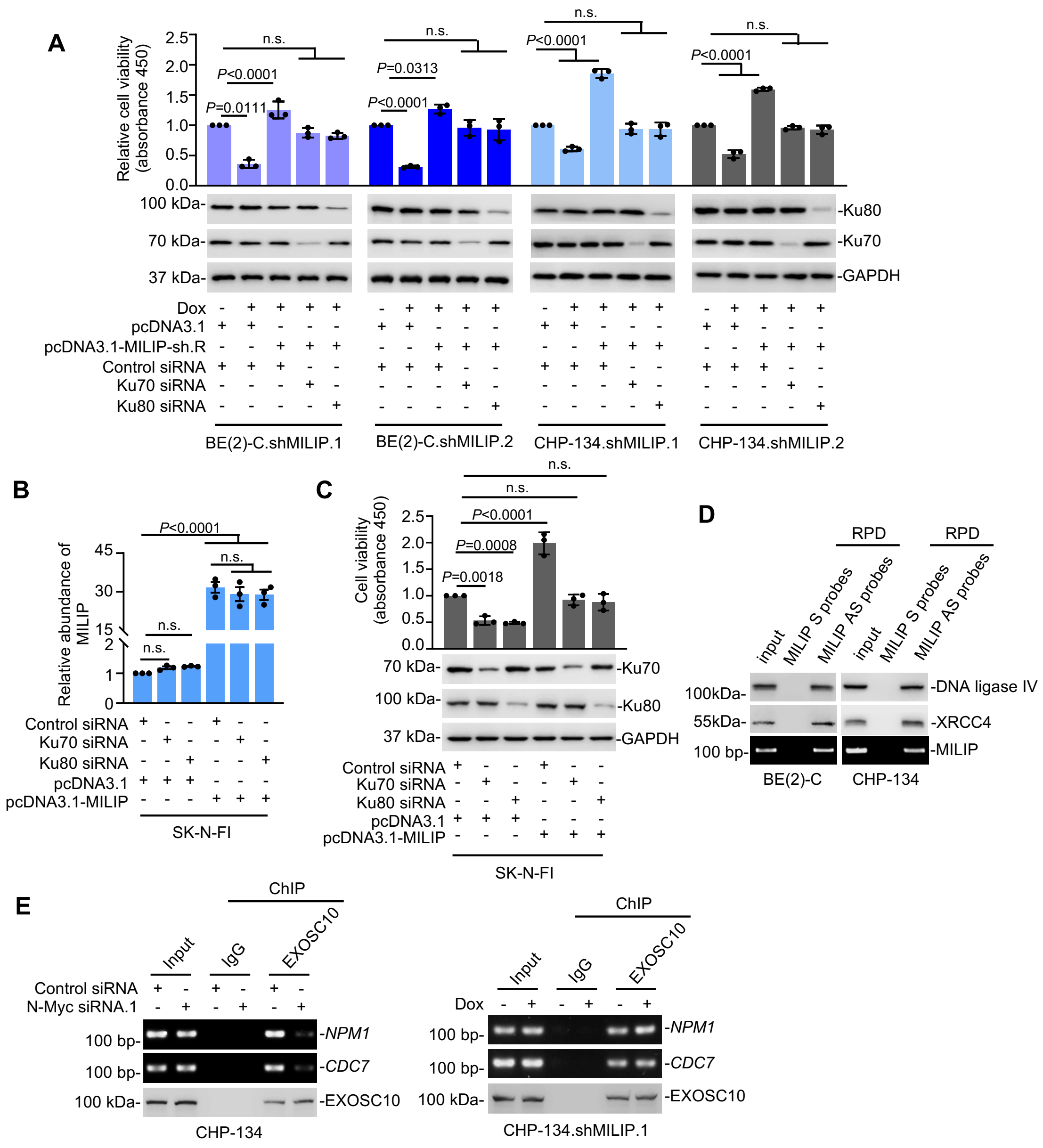

Fig. S6. MILIP protects neuroblastoma cells from DNA damage through Ku70 and Ku80. A, knockdown of Ku70 or Ku80 diminished the restoration of cell viability caused by introduction of MILIP-sh.R into BE(2)-C.shMILIP and CHP-134.shMILIP cells with endogenous MILIP knockdown by the addition of Dox (100ng/ml) (Data shown are mean ± SEM (upper) or representative (lower) of 3 independent experiments. One-way ANOVA followed by Tukey's multiple comparison). **B,** qPCR quantitation of overexpression of MILIP in SK-N-FI cells transfected with pcDNA3.1-MILIP (Data shown are mean ± SEM of 3 independent experiments. One-way ANOVA followed by Tukey's multiple comparison). **C**, knockdown of Ku70 or Ku80 abolished the increase in cell viability caused by MILIP overexpression in SK-N-FI cells (Data shown are mean ± SEM (upper) or representative (lower) of 3 independent experiments. One-way ANOVA followed by Tukey's multiple comparison). **D,** DNA ligase IV and XRCC4 were co-pulled down (RPD) by MILIP (Data shown are representative of 3 independent experiments). **E,** knockdown of N-Myc (left) but not knockdown of MILIP (right) reduced the association of EXOSC10 with the promoters of *NPM1* and *CDC7* as shown in ChIP assays (Data shown are representative of 3 independent experiments).

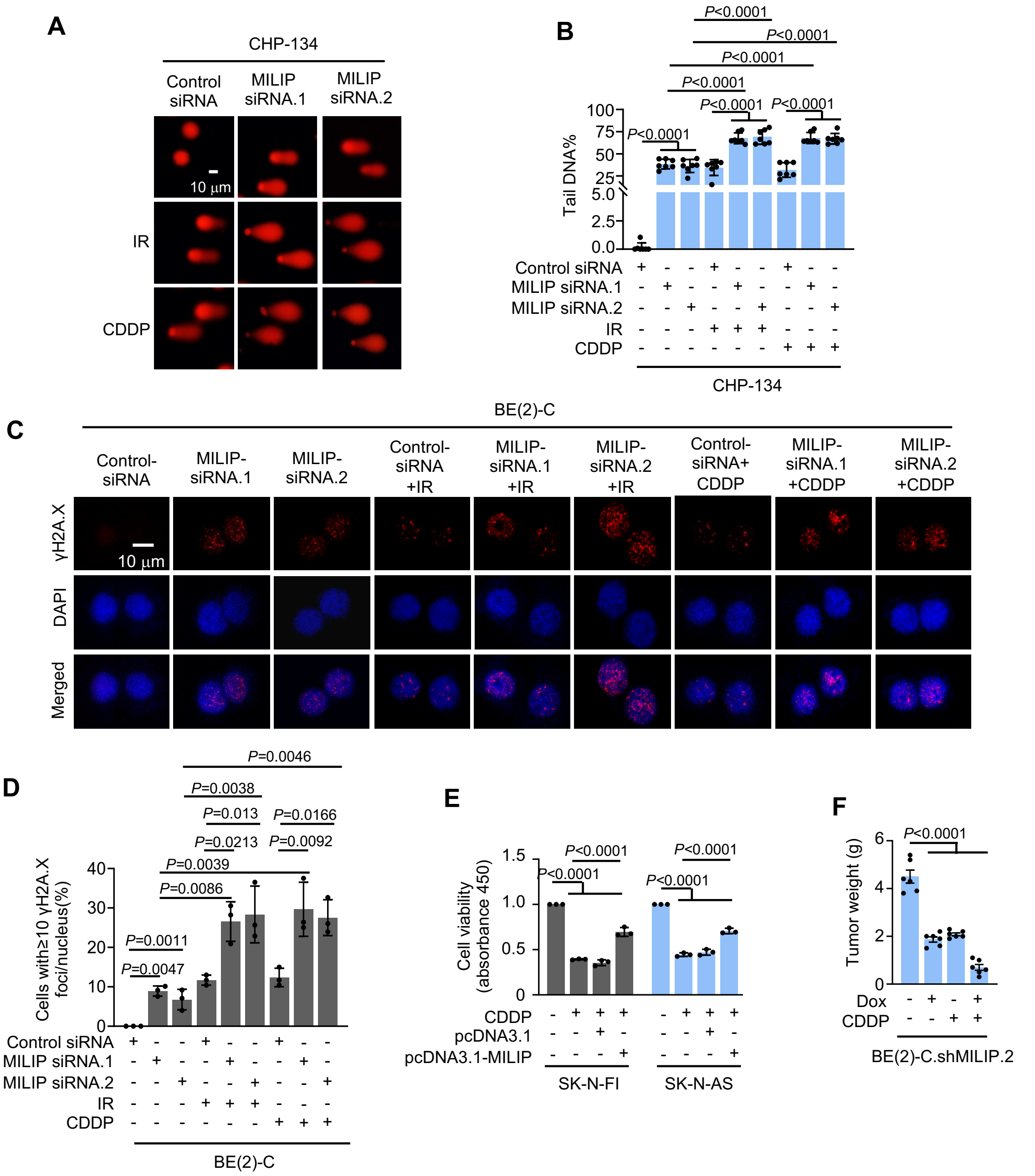

Fig. S7. MILIP protects neuroblastoma cells against DNA damaging therapeutics. A, siRNA knockdown of MILIP and treatment with ionizing radiation (IR, (10Gy, once) or CDDP (5mM for 24 hours) cooperatively induced the appearance of comet tails in CHP-134 cells (Data shown are representative of 3 independent experiments). **B**, quantitation of the relative tail DNA content of the comets as shown in A (Data shown are mean ± SEM of 3 independent experiments. One-way ANOVA followed by Tukey's multiple comparison). **C**, siRNA knockdown of MILIP and IR (10Gy, once) or CDDP (5mM for 24 hours) cooperatively induce DNA damage as shown by increased formation of γH2A.X foci (red) in BE(2)-C cells (Data shown are representative of 3 independent experiments). **D**, quantitation of the percentage of cells with γH2A.X foci as shown in C (Data shown are mean ± SEM of 3 independent experiments. One-way ANOVA followed by Tukey's multiple comparison). **E**, overexpression of MILIP attenuated reductions in cell viability caused by treatment with CDDP (5mM for 24 hours) in SK-N-FI and SK-N-AS cells (Data shown are mean ± SEM of 3 independent experiments. One-way ANOVA followed by Tukey's multiple comparison). **F**,

quantitation of tumor weights as shown in Figure 5E and F showing co-treatment with Dox (1 mg/ml supplemented with 10 mg/ml sucrose in drinking water) and CDDP (1mg/kg, i.p. injection) induced greater inhibition of BE(2)-C.shMILIP.2 xenograft growth than treatment with Dox or CCDP alone in nu/nu mice (n = 6 mice per group, mean ± SEM, one-way ANOVA followed by Tukey's multiple comparison).

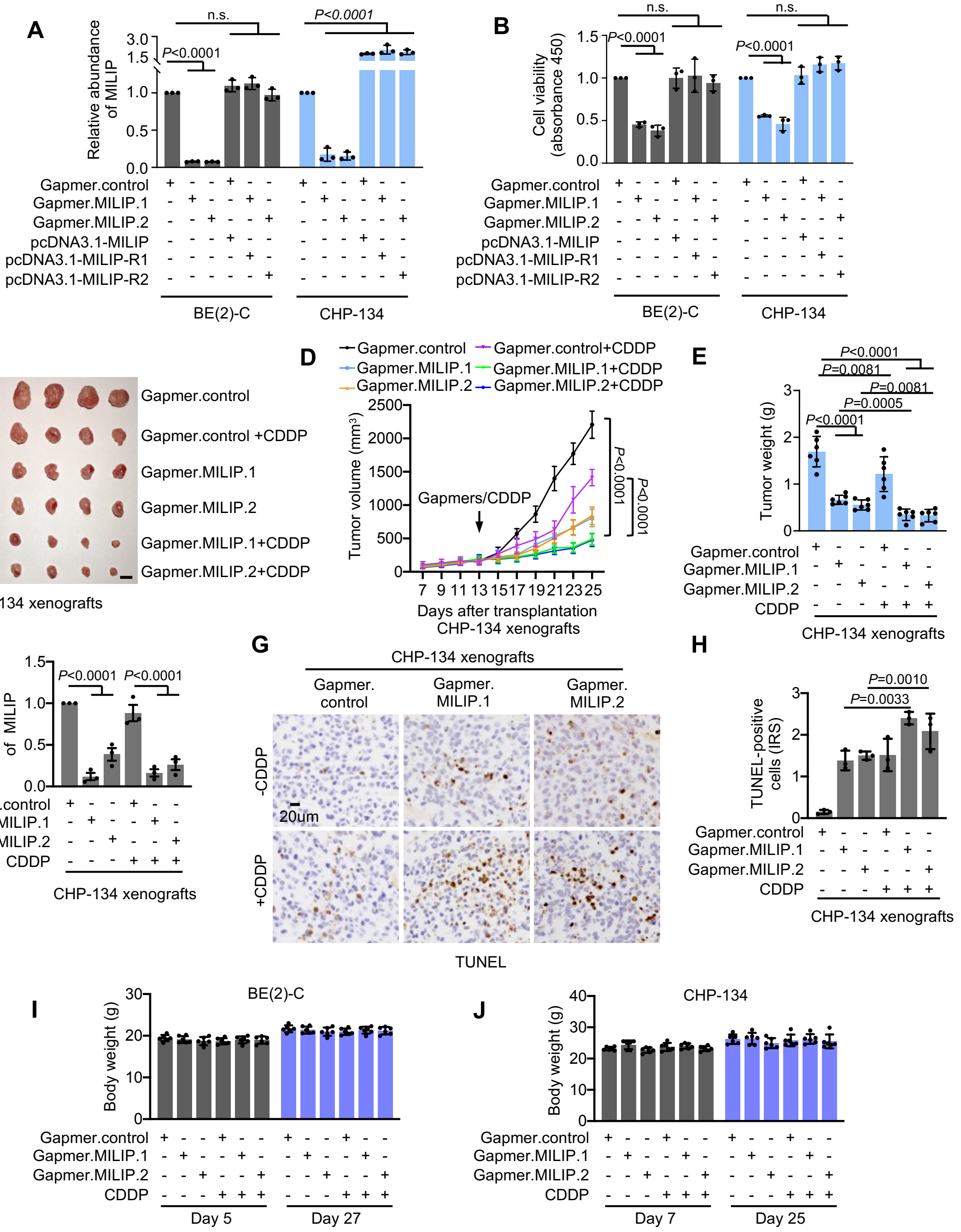

Fig. S8. Co-treatment with Gampers against MILIP (Gamper.MILIP1 and Gamper.MILIP2) and CDDP cooperatively inhibits neuroblastoma growth. A, quantitation of MILIP expression levels in BE(2)-C (C) and CHP-134 cells with or without co-transfection of Gapmer.MILIP and MILIP mutants (MILIP-R1 and MILIP-R2) carrying mismatches in the Gapmer.MILIP.1 and Gapmer.MILIP.2 targeting sequences, respectively (Data shown are mean ± SEM of 3 independent experiments. One-way ANOVA followed by Tukey's multiple comparison). **B**, co-transfection of MILIP mutants carrying mismatches in the Gapmer.MILIP.1 and Gapmer.MILIP.2 targeting sequences, respectively, rescued BE(2)-C and CHP-134 cells from viability reductions caused by Gapmer.MILIP (Data shown are mean ± SEM of 3 independent experiments. One-way ANOVA followed by Tukey's multiple comparison). **C**, photographs of CHP-134 xenografts in nu/nu mice treated as indicated (n = 6 mice per group). Scale bar, 1 cm. **D,** growth curves of CHP-134 xenografts in nu/nu mice treated as indicated (n = 6 mice per group. One-way ANOVA followed by

Tukey's multiple comparison). **E,** quantitation of tumor weights as in C showing that co-treatment with Gapmer.MILIP (10mg/kg, i.v. injection) and CDDP (1mg/kg, i.p. injection) induced greater inhibition of CHP-134 tumor growth than treatment with Gapmer.MILIP or CDDP alone in nu/nu mice (n = 6 mice per group, mean ± SEM, one-way ANOVA followed by Tukey's multiple comparison). **F,** MILIP expression in representative CHP-134 tumors (n = 3 tumors per group, mean ± SEM. One-way ANOVA followed by Tukey's multiple comparison). **G,** representative microscopic photographs of TUNEL staining on randomly selected tumor tissues from mice treated in C and D (n = 3 tumors per group. One-way ANOVA followed by Tukey's multiple comparison). **H,** quantitation of TUNEL staining as shown in G (n = 3 tumors per group, mean ± SEM. One-way ANOVA followed by Tukey's multiple comparison). IRS: Immunoreactive score. **I and J,** body weight of each mouse carrying BE(2)-C (I) or CHP-134 (J) was measured using a scale every other day commencing at day 5 (mice carrying BE(2)-C xenografts) or day 7 (mice carrying CHP-134 xenografts) after tumor cell implantation and the day when mice were sacrifices (n = 6 mice per group, mean ± SEM, one-way ANOVA followed by Tukey's multiple comparison).

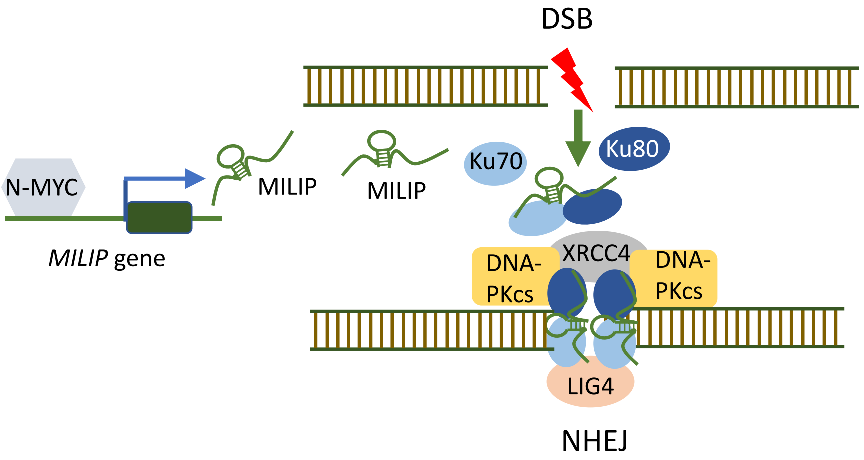

Fig. S9. A schematic model illustrating that N-Myc transcriptionally activates MILIP to promote the NHEJ pathway through facilitating the heterodimerization between Ku70 and Ku80 in neuroblastoma cells.

Table S1. Clinicopathological characteristics of the cohort of 27 neuroblastoma patients

Characteristics	Cases	MILIP abundance in Neuroblastoma (RS[a, b])	*P* value[c]
Gender	27		
	13	0.92 ± 0.30	*P*=0.556
Female	14	0.71 ± 0.20	
Age	27		
≥4[d]	13	0.96 ± 0.20	*P*=0.441
<4	14	0.68 ± 0.28	
INSS Stage[e]	27		
I /II	15	1.03 ± 0.26	*P*=0.160
III/IV	12	0.54 ± 0.18	

[a]RS: Reactive score; [b]Data shown are mean ± S.E.M.; [c]Student's *t*-test; a *P* value less than 0.05 was considered statistically significant; [d]The median age of the patients in this cohort was 4; [e]The median age of INSS Stage: International Neuroblastoma Staging System.

Table S2. Summary of MILIP-interacting proteins detected using mass spectrometry

No.	Entry name	Coverage (%)	MW (kDa)	Score
1	Ku70	56	69.8	233.19
2	HSP7C	18	70.9	33.19
3	PCCA	24	80	30.72
4	Ku80	18	82.7	25.82
5	IMP-3	18	63.7	21.78
6	PBP74	13	73.6	20.05
7	IMP-1	13	63.4	17.4
8	ATNUC-L1	15	76.6	16.82
9	PKM	22	57.9	16.81
10	CMC2	10	74.1	9.18
11	CARF	8	61.1	8.93
12	CK-16	13	51.2	8.48
13	PLAK	11	81.7	8.4
14	G4R1	3	114.7	6.57
15	PAP-IV	6	38.6	6.33
16	ARGRS	5	75.3	6.02
17	MCCA	6	80.4	5.77
18	PYC	3	129.6	5.51
19	DESP	3	331.6	4.67
20	RPN1	7	68.5	4.47
21	HBB	16	16	4.15
22	TCP4	16	14.4	3.94
23	H2A2C	12	14	3.83
24	NAA16	1	101.4	3.76
25	HLP2	6	73.2	3.74
26	PESC	3	68	3.54
27	H4	21	11.4	2.56
28	HSP76	5	71	2.53
29	ACC1	1	265.4	2.52
30	H13	5	22.3	2.43
31	GPAT	4	36	2.32
32	POTEF	1	121.4	2.3
33	TBA3D	2	49.9	2.24
34	DDX17	2	80.2	2.16
35	HNRNP A/B	3	36.2	2.16
36	RECQ1	4	73.4	2.14
37	CDHF4	5	113.7	2.1
38	EF1A2	2	50.4	2.1
39	ANS4B	3	46.6	2.09
40	DCD	10	11.3	2.06
41	ABCE1	2	67.3	1.93
42	RL8	4	28	1.9
43	SRP68	2	70.7	1.86
44	CK-80	2	50.5	1.85
45	H33	5	15.3	1.84
46	PTB	2	57.2	1.84

47	SEPT9	2	65.4	1.84
48	TIAM1	1	177.4	1.78
49	PRMT5	2	72.6	1.77
50	XRN2	1	108.5	1.77
51	TOM70	2	67.4	1.75
52	DDX41	1	69.8	1.72
53	RMI1	1	70.1	1.71
54	PAAF1	11	42.2	0
55	CH60	4	61	0
56	MED13	1	239.1	0
57	HNRNP	5	38.4	0
58	ENK11	8	21.4	0
59	SYK	2	68	0

Table S3. Information on the cell lines used in this study

Cell lines	Source	Catalogue No.	RRID	Culture Conditions
BE(2)-C	ATCC	CRL-2268	CVCL_0529	DMEM[a] with 10%FBS[b]
CHP-134	Creative Bioarray	CSC-C0635	CVCL_1124	RPMI-1640[c] with 10%FBS
SK-N-AS	ATCC	CRL-2137	CVCL_1700	DMEM with 10%FBS, 1% NEAA[d]
SK-N-FI	ATCC	CRL-2142	CVCL_1702	DMEM with 10%FBS, 1% NEAA
HEK293	National Collection of Authenticated Cell Cultures	SCSP-502	CVCL_0045	DMEM with 10%FBS

[a]DMEM: Dulbecco's modified Eagle's medium (DMEM) (Biological Industries, 2152423, Beit Haemek, Israel)
[b] FBS: Fetal bovine serum (Biological Industries, 04-001-1A, Beit Haemek, Israel)
[c]RPMI-1640: Roswell Park Memorial Institute Medium (RPMI) 1640 (Biological Industries, 2145238, Beit Haemek, Israel)
[d] NEAA: Non-essential amino acids (Zqxzbi, CSP008, Shanghai, China)

Table S4. List of antibodies

Antibody (Ab)	Catalogue No.	Company	Dilution
N-Myc Rabbit pAb[a]	10159-2-AP	Proteintech Group (Wuhan, Hubei, China)	1:50-1:500
Ku70 Mouse mAb[b]	66607-1-Ig	Proteintech Group (Wuhan, Hubei, China)	1: 2500
Ku70 rabbit pAb	10723-1-AP	Proteintech Group (Wuhan, Hubei, China)	1:100
Ku80 Mouse mAb	66546-1-Ig	Proteintech Group (Wuhan, Hubei, China)	1:2500
Normal Mouse IgG pAb	sc-2025	Santa Cruz Biotechnology (Dallas, TX)	1: 500
Normal rabbit IgG mAb	ab172730	Abcam (Cambridge, UK)	1:1000
Ki67 Rabbit pAb	27309-1-AP	Proteintech Group (Wuhan, Hubei, China)	1:10000
β-actin Mouse pAb	66009-1-Ig	Proteintech Group (Wuhan, Hubei, China)	1:10000
H2A.X mAb	D7T2V	Cell Signaling Technology (Shang Hai, China)	1:100
53BP1 Rabbit pAb	4937	Cell Signaling Technology (Shang Hai, China)	1:100
p53 pAb Rabbit pAb	10442-1-AP	Proteintech Group (Wuhan, Hubei, China)	1:1000
p-p53 （Ser15） Mouse mAb	9286	Cell Signaling Technology (Shang Hai, China)	1:1000
ATM Rabbit mAb	ab32420	Abcam (Cambridge, UK)	1:3000
p-ATM (T68) Rabbit mAb	ab81292	Abcam (Cambridge, UK)	1:50000
Chk2 Rabbit mAb	ab109413	Abcam (Cambridge, UK)	1:1000
p-Chk2 (Thr68) Rabbit pAb	2661	Cell Signaling Technology (Shang Hai, China)	1:1000
Exosc10 Rabbit pAb	16731-1-AP	Proteintech Group (Wuhan, Hubei, China)	1:10000
XRCC4 Rabbit pAb	15817-1-AP	Proteintech Group (Wuhan, Hubei, China)	1:1000
DNA ligase IV	ab193353	Abcam (Cambridge, UK)	1:1000
Caspase-3 Rabbit mAb	19677-1-AP	Proteintech Group (Wuhan, Hubei, China)	1:1000
PARP1 Rabbit pAb	13371-1-AP	Proteintech Group (Wuhan, Hubei, China)	1:1000
GAPDH Mouse pAb	60004-1-Ig	Proteintech Group (Wuhan, Hubei, China)	1:10000
HRP Conjugated AffiniPure Goat Anti-mouse IgG (H+L)	BA1050	BOSTER Biological Technology (Wuhan, Hubei, China)	1:2000

HRP Conjugated AffiniPure Goat Anti-rabbit IgG (H+L)	BA1054	BOSTER Biological Technology (Wuhan, Hubei, China)	1:2000
DyLight 488-Goat anti-Mouse IgG (H+L)	BA1126	BOSTER Biological Technology (Wuhan, Hubei, China)	1:200
CY3-Goat anti-Ribbit IgG (H+L)	BA1032	BOSTER Biological Technology (Wuhan, Hubei, China)	1:200

[a]pAb: polyclonal antibody; [b]mAb: monoclonal antibody

Table S5. List of reagents

Reagent	Catalogue No.	Company
Doxycline	HY-N0565B	Med Chem Express (Nanjing, Jiangsu, China)
TRIzol^{TM} Reagent	15596018	Thermo Fisher Scientific (Waltham, MA)
Protease Inhibitor Cocktail	4693132001	Sigma-Aldrich (MERCK, Beijing, China)
RiboLock RNase Inhibitor	EO0384	Thermo Fisher Scientific (Waltham, MA)
4% paraformaldehyde	AR1068	BOSTER Biological Technology (Wuhan, Hubei, China)
Opti-MEM^{TM} Reduced Serum Medium	31985070	Thermo Fisher Scientific (Waltham, MA)
DNA extraction buffer	P1012	Solarbio Life Sciences (Beijing, China)
Protease K	P1120	Solarbio Life Sciences (Beijing, China)
z-VAD-fmk	HY-16658B	Med Chem Express (Nanjing, Jiangsu, China)

Table S6. List of primers and RNA pulldown probes

qRT-PCR primers	MILIP	Forward: AGAACCGCGAAAGGCTACTG<br>Reverse: CACTTAAAGCCGGTCGTGGA
	N-Myc mRNA	Forward: GCGTCGCAGAAACCACAAC<br>Reverse: CAGCAGCAGTTGCTAAAGA
	GAPDH	Forward: GCTCTCTGCTCCTCCTGTTC<br>Reverse: ACGACCAAATCCGTTGACTC
	β-actin	Forward: GGACTTCGAGCAAGAGATGG<br>Reverse: AGCACTGTGTTGGCGTACAG
	18S	Forward: GCTTAATTTGACTCAACACGGGA<br>Reverse: AGCTATCAATCTGTCAATCCTGTC
	U6	Forward: TCGCTTCGGCAGCACATAT<br>Reverse: ATTTGCGTGTCATCCTTGC
	PVT1	Forward: TGAGAACTGTCCTTACGTGACC<br>Reverse: AGAGCACCAAGACTGGCTCT
RT-PCR primers (*in vitro* transcription)	MILIP	Forward: TAATACGACTCACTATAGGGATGCGCAACCCGCGCGCCGT<br>Reverse: TCCCCAGCAGGCCAGGTGGGC
	MILIP-△E1	Forward: TAATACGACTCACTATAGGGAGAGAGCTGAAGGTGTTCCGT<br>Reverse: TCCCCAGCAGGCCAGGTGGGC
	MILIP -△E2	Forward: TAATACGACTCACTATAGGGATGCGCAACCCGCGCGCCGT<br>Reverse: GCTGGCGGGGCGGCCACCCTTC
	MILIP-△E2-991/-1895	Forward: TAATACGACTCACTATAGGGATGCGCAACCCGCGCGCCGT<br>Reverse: GGATGCCCGGGAGGTGCCTG
	MILIP-△E2-1489/-1895	Forward: TAATACGACTCACTATAGGGATGCGCAACCCGCGCGCCGT<br>Reverse: CTTGCCAGCTGGGGCCCTTGC
RT-PCR Primers (ChIP)	NPM1	Forward: TTCACCGGGAAGCATGG<br>Reverse: CACGCGAGGTAAGTCTACG
	CDC7	Forward: TTTCCTGACTTCAAACCGCC<br>Reverse: GGGCTGCAGGGAGAATTT
pcDNA3.1(+)-MILIP-sh.R		Forward1: GCTCTAGAATGCGCAACCCGCGCGC<br>Reverse1: CTTGGTTGGAAGCGCTCTTGATCCG<br>Forward2: CTGGATCAAGAGCGCTTCCAACCAG<br>Reverse2: CCCAAGCTTCCCCAGCAGGCCAGGTGGG
MILIP-biotin-probes		AS[a]1: TGACCACGGAACACCTTCAG<br>AS2: ACTGTGTCCAGGGACAAGTG<br>AS3: TGAGAGGGATGCTTGGAAACC<br>S[b]1: GACAATACTCGACAGGCTCC<br>S2: GAGCGAAGGTTATGTCGACC

S3: GTGAACAACGCGAGTTGTGGA

[a]AS: antisense; [b]S: sense.

Table S7. List of target sequences of siRNAs/shRNAs

siRNAs/ shRNAs/ sgRNA	MILIP	siRNA.1/shRNA.1: GGAGUCAGGGCAAUUCCAA<br>siRNA.2/shRNA.2: GGUAACAUAGAGACCCUAU
	N-Myc	siRNA.1: CCUCGAGUUUGACUCGCUA<br>siRNA.2: CAGCAGCAGUUGCUAAAGA
	Ku70	siRNA.1: GUGAUGUCCAAUUCAAGAU<br>siRNA.2: GCAUCUUCCUUGACUUGAU
	Ku80	siRNA.1: GGCCUUCCUUUCCACUAAA<br>siRNA.2: GGCUCCAAUUUGUCUAUAA
	P53 sgRNA	sgRNA-F: CACCGGGCAGCTACGGTTTCCGTC<br>sgRNA-R: AAACGACGGAAACCGTAGCTGCCC

Table S8. Treatment protocols of xenograft mouse model

Medicine	Treatment routes	Concentrations	Solvents	Volume	Frequency
Gapmer-control	i.v.[a]	10 mg/kg	saline	100µL	Every other day (10times)
Gapmer.MILIP1	i.v.	10 mg/kg	saline	100µL	Every other day (10times)
Gapmer.MILIP2	i.v.	10 mg/kg	saline	100µL	Every other day (10times)
Cisplatin	i.p.[b]	1 mg/kg	saline	100µL	Every other day (10 times)
Doxycycline	i.p.	2 mg/kg	saline	150µL	Every other day (7 times)

[a]i.v.: Intravenous; [b]i.p.: Intraperitoneal.
